# Proceedings of National Initiative VI

**DOI:** 10.31486/toj.NI2020

**Published:** 2020

## Why a National Initiative?

Both the public and our profession acknowledge that quality and safety efforts are falling short, and many hospitals and healthcare systems are seeking rapid improvements in patient care. Those of us in academic medicine realize that residents play an important role in patient care at teaching institutions; however, residents are generally not visible in safety and quality efforts. The Alliance of Independent Academic Medical Centers (AIAMC) recognized that resident quality improvement efforts—shared across multiple programs and systems—had the potential to improve care quickly and effectively.

## Role of the AIAMC

The AIAMC was founded in 1989 as a national network of large academic medical centers. Membership in the association is unique in that AIAMC members are affiliated with medical schools but are independent of medical school ownership or governance. Ninety major medical centers and health systems across the United States are members, representing more than 750 senior academic leaders.

## National Initiative I

In early 2007, the AIAMC launched *Improving Patient Care Through GME: A National Initiative of Independent Academic Medical Centers*. The National Initiative (NI) featured 5 meetings over the course of 18 months that served as touchstones for ongoing quality improvement in 19 AIAMC participating organizations. These meetings, as well as the monthly collaborative calls held in between, provided structure, discussion, and networking opportunities around specific quality improvement initiatives. NI I was supported by a grant from the foundation of HealthPartners Institute for Medical Education, an AIAMC member institution located in Minneapolis, MN. As a result of these efforts, we developed initial findings that demonstrated the efficacy of integrating GME into patient safety and quality improvement initiatives. These findings were organized into a series of articles that were published in the December 2009 issue of *Academic Medicine*.

## National Initiative II

In 2009, we launched NI II and expanded participation to 35 AIAMC-member teaching hospitals from Seattle to Maine. Each participating hospital developed a quality improvement team led by a resident or faculty member. These teams met onsite 4 times and participated in monthly conference calls over an 18-month period. Quality improvement projects focused on one of the following areas: communication, handoffs, infection control, readmissions, and transitions of care. Results from NI II were published in a variety of publications, including the February 2011 issue of the *AAMC Reporter*, and in the May/June 2012 special supplement issue of the *American Journal of Medical Quality*.

## National Initiative III

NI III, launched in 2011 with 35 teams, built on the strengths of the first 2 NIs and moved beyond direct support of local quality improvement teams to the development of teaching leadership and changing organizational culture to support quality improvement initiatives. Graduate medical education *and* continuing medical education were emphasized as platforms for improving patient care. The focus of NI III was faculty/leadership development. We recognized that part of our responsibility as medical educators was to train the next generation of practicing physicians; thus, residents must be considered as junior faculty and were integral in this effort. Results from NI III were published in a variety of publications, including the Spring 2014 issue of the *Ochsner Journal* and the *Journal of the American College of Surgeons.*

## National Initiative IV

NI IV: *Achieving Mastery of CLER*, launched in 2013 with 34 AIAMC-member and—for the first time—nonmember teams, focused on navigating the ACGME's Clinical Learning Environment Review (CLER) program. The CLER program was designed to evaluate the level of institutional responsibility for the quality and safety of the learning and patient care environment, and NI IV provided teams the training and guidance necessary to identify strengths and weaknesses across the 6 focus areas and significantly and measurably advance the institutional level of preparedness. Results from NI IV were published in numerous publications, including the *Journal of Graduate Medical Education* and the *Ochsner Journal*, the official publication of the AIAMC National Initiatives.

## National Initiative V

NI V: *Improving Community Health and Health Equity Through Medical Education* launched in fall 2015 with 29 AIAMC member teams participating and focused on navigating the disparities component of the ACGME's Clinical Learning Environment program. Four onsite learning sessions addressed understanding and engaging with institutional leaders in the Community Health Needs Assessments; graduate medical education in improving health equity, cultural competency, and community engagement; and how to better engage the C-suite. NI V concluded in March 2017. Results from NI IV were published in numerous publications, including the *Journal of Graduate Medical Education* and the *Ochsner Journal*, the official publication of the AIAMC National Initiatives.

## National Initiative VI

NI VI: *Stimulating a Culture of Well-Being in the Clinical Learning Environment* launched in fall 2017 with 34 AIAMC member teams participating. Teams were grouped into cohorts based upon similarities of projects in the following domains: culture and values; institutional well-being; meaning in work, work-life integration and social support and community at work; and workload and job demands and control and flexibility. The Initiative concluded in March 2019 at the fourth and final meeting where teams presented their concluding posters and outcomes. Various writing teams are currently preparing manuscripts for publication.

***The AIAMC National Initiative is the only national and multiinstitutional collaborative of its kind in which residents lead multidisciplinary teams in quality improvement projects aligned to their institution's strategic goals. Sixty-four hospitals and health systems and more than 1,000 individuals have participated in the AIAMC National Initiatives since 2007 and have driven change that resulted in meaningful and sustainable outcomes that improved the quality and safety of patient care. For more information, visit aiamc.org/national-initiative.***

**Kimberly Pierce-Boggs**

AIAMC Executive Director

www.aiamc.org

kimberly@aiamc.org

312-836-3712

## ALLIANCE OF INDEPENDENT ACADEMIC MEDICAL CENTERS BOARD OF DIRECTORS

**Ronald G. Amedee, MD**

Designated Institutional Official

Ochsner Health System

**Laurinda Calongne, EdD**

Chief Academic Officer

Designated Institutional Official

Our Lady of the Lake Regional Medical Center

**Lynne A. Chafetz, JD**

Senior Vice President and General Counsel

Virginia Mason Medical Center

**Eric Crowell, MAH**

President and CEO

UnityPoint Health – Des Moines

**Robert Dressler, MD, MBA**

Quality and Safety Officer, Academic

and Medical Affairs

Christiana Care Health System

**Judith A. Gravdal, MD**

Chair, Family Medicine

Advocate Lutheran General Hospital

**Kevin T. Hinchey, MD**

Chief Education Officer

Baystate Health, Inc.

**Joseph Jaeger, DrPH**

Chief Academic Officer

Barnabas Health – Monmouth Medical Center

**Hania Janek, PhD**

Vice President, Education

Baylor Scott & White

**David S. Kountz, MD**

Co-Chief Academic Officer

Hackensack Meridian Health – Jersey Shore

University Medical Center

**Joyce Lammert, MD**

Designated Institutional Official; GME; Executive

Medical Director, Hospital

Virginia Mason Medical Center

**Barry D. Mann, MD**

Chief Academic Officer

Main Line Health System

**Tsveti Markova, MD**

Designated Institutional Official

Ascension Providence Rochester Hospital

**Shelly Monks, MBA, FACHE**

System Vice President, Academic Affairs

Ochsner Health System

**James Orlando, EdD**

Associate Chief Academic Officer

Designated Institutional Official

St. Luke's University Health Network – University

Hospital Bethlehem

**Joseph Portoghese, MD**

Chief Academic Officer

Designated Institutional Official

AdventHealth Orlando

**Heather Sankey, MD**

Chair, Department of OB/GYN

Baystate Health, Inc.

**Deborah Simpson, PhD**

Medical Education Program Director

Aurora Health Care

## ALLIANCE OF INDEPENDENT ACADEMIC MEDICAL CENTERS COMMITTEE ON THE INTEGRATION OF ACADEMICS AND QUALITY – NATIONAL INITIATIVE VI

**Barry Mann, MD, Chairman**

Main Line Health System

**Lynne Chafetz, JD, Vice-Chairman**

Virginia Mason Medical Center

**Judith A. Gravdal, MD**

Advocate Lutheran General Hospital

**Steve Johnson, MD**

TriHealth

**David S. Kountz, MD**

Hackensack Meridian Health – Jersey Shore

University Medical Center

**Tim Lineberry, MD**

Aurora Health Care

**Deborah Simpson, PhD**

Aurora Health Care

**William (John) Yost, MD**

UnityPoint Health – Des Moines

**Kimberly Pierce-Boggs**

Alliance of Independent Academic Medical Centers

## ALLIANCE OF INDEPENDENT ACADEMIC MEDICAL CENTERS National Initiative VI Participating Institutions and Team Leaders

**AdventHealth – Orlando (Florida Hospital)**

Joseph Portoghese, MD

**Advocate Health Care – Advocate Illinois Masonic Medical Center**

Mohammed Samee, MD

**Advocate Health Care – Advocate Lutheran General Hospital**

Judith A. Gravdal, MD

**Arrowhead Regional Medical Center**

Teresa Smith, MBA

**Ascension Providence Rochester Hospital (Crittenton Hospital Medical Center)**

R. Brent Stansfield, PHD

Tsveti Markova, MD, FAAP

**Atrium Health – Carolinas Medical Center**

Eric Anderson, MEd

Suzette Caudle, MD

**Aurora Health Care**

Jacob Bidwell, MD

Deborah Simpson, PhD

**Bassett Medical Center**

James Dalton, MD

**Baylor University Medical Center at Dallas**

Cristie Columbus, MD

**Baystate Health**

Kevin Hinchey, MD

Heather Sankey, MD

**Billings Clinic**

Virginia Mohl, MD, PhD

**Cedars-Sinai Medical Center**

Betsy McGaughey

Mark Noah, MD

**Christiana Care Health System**

Vanessa Downing, PhD

**Cleveland Clinic – Akron General Medical Center**

Cheryl Goliath, PhD

**Community Health Network**

Jesse Clark, DO

Kathy Zoppi, PhD, MPH

**Guthrie – Robert Packer Hospital**

John Pamula, MD

**Hackensack Meridian Health – Jersey Shore**

**University Medical Center**

Paul Schwartzberg, DO

**HCA South Atlantic Division**

John Lucas, DO

Lori McMann, MS

**HealthPartners Institute**

Cullen Hegarty, MD

**HonorHealth**

Wendy Ellis, MC, LPC, DHEd

**Main Line Health System**

Joseph Greco, MD, FAAFP

**Maine Medical Center**

Kalli Varaklis, MD, MSEd

**Monmouth Medical Center – RWJBarnabas Health**

Joseph Jaeger, DrPH

**Ochsner Health System**

Stewart Hart, MD

**OhioHealth – Riverside Methodist Hospital**

Stephen Auciello, MD

**Orlando Health**

Kwabena Ayesu, MD

**OSF Healthcare**

Francis McBee-Orzulak, MD

**Our Lady of the Lake Regional Medical Center**

Rebecca Horn, PhD

**Saint Francis Hospital and Medical Center**

Jeri Hepworth, PhD

**Sinai Hospital of Baltimore**

Diane Maloney-Krichmar

Asha Thomas, MD

**The Christ Hospital Health Network**

Jennifer Reemtsma, MEd

**TriHealth**

Dave Dhanraj, MD

**UnityPoint Health – Des Moines**

Chanteau Ayers, JD

**Virginia Mason Medical Center**

Gillian Abshire, RN

## ABBREVIATIONS/ACRONYMS

ACGME – Accreditation Council for Graduate Medical Education

AHA – American Heart Association

ANOVA – Analysis of variance

CDC – Centers for Disease Control and Prevention

CEO – Chief executive officer

CG-CAHPS – Clinician and Group Consumer

Assessment of Healthcare Providers and Systems

CLER – Clinical Learning Environment Review

CMO – Chief medical officer

DIO – Designated institutional official

EAP – Employee assistance program

EHR – Electronic health record

EMR – Electronic medical record

FTE – Full-time equivalent

GME – Graduate medical education

GMEC – Graduate Medical Education Committee

ICU – Intensive care unit

IHI – Institute for Healthcare Improvement

IT – Information technology

OB/GYN – Obstetrics and gynecology

PDSA – Plan, Do, Study, Act

PGY – Postgraduate year

QI – Quality improvement

## AdventHealth (Florida Hospital), Orlando, FL

### Flourish Through CREATION Health

#### Joe Portoghese, MD; Amanda Sawyer, PhD; Patricia Robinson, PhD; Kathy Gibney, PhD, APBB; Leigh DeLorenzi, PhD, LMHC; Mandi Bailey, MA, LMHC; Serena Gui, PhD; Yvette Saliba, PhD

##### 

###### Background:

Previous studies have shown an association between positive peer relationships, self-care workshops, and mindfulness-based practices with reduced burnout in residency. AdventHealth GME and the Center for Physician Wellbeing developed a new well-being initiative for residents based on their existing whole person wellness philosophy of CREATION Health. CREATION is an acronym for choice, rest, activity, trust, interpersonal relationships, outlook, and nutrition. The new program, named Flourish Through CREATION Health, was grounded in research and funded by the Center for CREATION Health Research.

###### Methods:

One hundred sixty-seven residents in all GME programs participated in six 90-minute, small-group sessions and two booster sessions. These sessions involved various components such as expressive arts, mindful living practices, and self-reflection. Online survey packages were administered at baseline, midpoint, postprogram, and 3-month follow-up. Included in the survey packages were the Maslach Burnout Inventory (MBI), the Perceived Stress Scale, the Life Satisfaction Survey, and the Resident Wellness Scale. A paper feedback form was also administered during the final group session to gauge participants' experience and whether they found the sessions helpful.

###### Results:

The mean age of participants was 30.8 (SD 4.1) years, with 39 PGY1 residents, 45 PGY2 residents, 39 PGY3 residents, 6 PGY4 residents, and 7 PGY5 residents. On the MBI, 58.2% of residents reported that they had not experienced emotional exhaustion/depersonalization compared to 29.1% who reported these symptoms. The mean score on the Perceived Stress Scale [ranges from 0 (low) to 40 (high)] was 15.2 (SD 7.2), and the mean score on the Resident Wellness Scale [ranges from 0 (low) to 4 (high)] was 2.8 (SD 0.7). On the program evaluation feedback form, more than half of the participants agreed that their overall well-being had improved, trust among their peers improved, and new information about communication was learned.

###### Conclusion:

The program resulted in improvement in trust and communication skills among residents, but the attendance mandate was a barrier to engagement. For continued improvement among residents, a menu of various options for well-being interventions that can be chosen by residents or programs to meet the institutional goal of integrating well-being is being developed. Booster sessions called Resilience Rounds will also continue.

**Table:** PROJECT MANAGEMENT PLAN – Flourish Through CREATION Health

Vision Statement	Our vision is to build resilience in residents through experiential small-group activities grounded in the CREATION Health principles of choice, rest, environment, activity, trust, interpersonal relationships, outlook, and nutrition. In addition to providing all residents with a space for personal exploration, this initiative will examine whether participation in these small groups influences resident burnout, perceived stress, empathy, and/or well-being.
Team Objectives	Through their participation, AdventHealth residents will be able to understand the 8 CREATION Health principles of well-being with an emphasis on choice, trust, interpersonal relationships, and outlook build trust and enhance communication skills practice strategies designed to reinforce resiliency skills, including internal reflection, examining emotions, expanding choices, deepening empathy, reclaiming personal meaning, and enriching relationships
Success Factors	The most successful parts of our work were the outcome of improved trust among residents and the reported appreciation of the small-group format. We were inspired by the Center for Physician Wellbeing's improved relationships with residents, as well as the increased awareness of and access to our services. We were also inspired by resident reactions to the content of the curriculum and direct feedback on how to improve the program to best meet their needs.
Barriers	The largest barrier encountered was that the Center for Physician Wellbeing designed and delivered the program but is a department outside, resulting in unclear roles, responsibilities, and ownership of the project among partners. Participants experienced survey fatigue and confusion about when to complete assessments. We worked to overcome these barriers by creating an advisory board consisting of faculty members, residents, facilitators, program developers, and the GME chief academic officer. We also increased communication with the research team and added time to final sessions to complete surveys.
Lessons Learned	The single most important piece of advice to provide another team embarking on a similar initiative is to make sure that the well-being/resiliency interventions are supported by and driven from within GME (leadership, faculty, and/or residents). Do not approach well-being initiatives as a manualized, one-size-fits-all approach.

## Advocate Illinois Masonic Medical Center, Chicago, IL

### Examining the Impact of a Support Group on Burnout and Resilience in Graduate Medical Trainees

#### Faizan Chaudhary Ahmed, MD; Marion Gonzalez, MD; Agness Gregg; Chris Kabir, MS; Gina Schueneman, DO; Amy Portacci, DO; Daniel Armbrust, DO; Leah Delfinado, MD; Maggie Pham, DO; Monica Lai, MD; Elizabeth Rutha, PsyD; Mohammed Samee, MD, RN, FACP

##### 

###### Background:

Residency programs have a strong commitment to address physician well-being in the clinical learning environment. Resident-physician support groups are a well-documented and accepted method to mitigate resident burnout and improve resilience. The aim of this project was to establish and sponsor resident support groups at one metropolitan hospital across three departments: family medicine (FM), OB/GYN, and internal medicine (IM).

###### Methods:

In this quasi-experimental, multidepartment, prospective cohort study, support group content focused on skill-building around coping and prioritizing values; processing the emotional challenges inherent in the work; and mindfulness-based stress reduction. IM residents (7 groups of 8 residents) had a 90-minute session each month; OB/GYN residents (2 groups of 6 residents) had a 60-minute session every 6 months; and FM residents (3 groups of 8 residents) had a 45-minute session every month. Facilitators were behavioral health faculty for OB/GYN and FM and behavioral health postgraduate fellows for IM. Survey tools included the Maslach Burnout Inventory, the Connor-Davidson Resilience Scale 25, and an Advocate Illinois Masonic Medical Center Support Survey, which was developed to examine overall experience and satisfaction every 6 months.

###### Results:

No significant differences were found between baseline and final follow-up among combined departments for emotional exhaustion, depersonalization, personal achievement, or resilience. The most commonly reported themes for helpful support group aspects were peer support and engagement (n=22) and decompression/venting frustration/talking about personal issues (n=11). Overall helpfulness differed significantly between FM, OB/GYN, and IM (*P*=0.05). Residents who thought the support groups were more helpful were more satisfied with the overall structure, frequency, time length, and quality of the meeting content. Residents who thought they had access to enough resources had higher resilience (*P*=0.10). IM was least satisfied with the time length of meetings (*P*<0.05). FM was most satisfied with resources dedicated to conduct meetings (*P*<0.05).

###### Conclusion:

We found no generalized effect of a support group intervention. Key differences were discovered between departments and within IM. Within the IM department, depersonalization changed as the residents progressed through the program, highlighting the need for interventions at 6 months. Facilitation of the support group for IM changed in September, which may have affected rapport and comfort with an established facilitator. Residents had high satisfaction with no more than an hour-long session. Continued steps should include small-group review to identify ideal length, structure, and content; a customized toolbox to allow residents to self-select appropriate strategies; and partnership with a wider wellness committee within the organization.

**Table:** PROJECT MANAGEMENT PLAN – Examining the Impact of a Support Group on Burnout and Resilience in Graduate Medical Trainees

Vision Statement	Our vision is to create a culture in the clinical learning environment such that resident physicians and those supporting resident training are well versed in identifying and managing available resources to address and promote well-being.
Team Objectives	Our primary aim was to establish and sponsor resident support groups at one metropolitan hospital across three departments: family medicine, OB/GYN, and internal medicine. We aimed to establish a safe atmosphere to process professional challenges, develop coping skills to manage burnout, and create a template for work-life balance and healthy lifestyle choices.
Success Factors	The most successful parts of our work were the implementation of support groups and the increased awareness among faculty and GME. We were inspired by the engagement of our residents and their willingness to participate.
Barriers	The largest barrier encountered was finding protected time for residents to participate and the lack of a one-size-fits-all support group. We worked to overcome this barrier by planning to have small-group review to identify ideal length, structure, and content.
Lessons Learned	The single most important piece of advice to provide another team embarking on a similar initiative is to include a customized toolbox to allow residents to self-select appropriate strategies.

## Advocate Lutheran General Hospital, Park Ridge, IL

### Well-Being in Our Family Medicine Residency: Curricular Content and Experiences

#### J Gravdal; K Koo; N Pagoria; P Piper; H Razzaq

##### 

###### Background:

Residency is an opportune time to prepare residents with skills to assess and foster their own well-being and influence the organizations in which they work. Our goals were to increase awareness of the importance of faculty and resident well-being and to implement a curriculum to improve knowledge of the importance of intentional focus on wellness and the development of lifelong skills.

###### Methods:

Interventions were directed at the family medicine residency, the organization (hospital and system), and the Advocate Lutheran General Hospital medical staff. Interventions and changes were the institution of a Family Medicine Residency Wellbeing Committee, curriculum development, and implementation of the curriculum. Measures/metrics for the family medicine residency included retreats, wellness Wednesdays, a self-care policy, Wheel of Life (an individual and program tool), and huddle tools. Measures/metrics for the hospital medical staff included a Physician Wellness Committee and Physician Wellness Week, and for the organization, a System Wellness Committee was a metric. The curriculum developed involved didactics, experiential learning (retreats, narrative medicine/storytelling, Mayo Wellbeing Index, and Wheel of Life), and other resources.

###### Results:

Family medicine residents participated, and progress was made toward a curriculum. Physician Wellness Week (which included residents) was held, and the third measure (organization) resulted in a System Wellness Committee and the appointment of a system GME Director of Well-Being, Academic Affairs.

###### Conclusion:

Resident and faculty well-being is important at many levels; the system recognizes this importance. Individuals have unique needs and perceptions. Multiple overlapping initiatives among different departments have similar goals but operate in silos. Challenges included transitions in and lack of support from the research department, system merger, an Epic transition, and departmental silos. Unintentionally, our work defaulted to family medicine only. Another limitation was competing equally important initiatives for team members' time and energy. The development and evaluation of the family medicine curriculum and collaboration with other residencies should continue.

## Advocate Lutheran General Hospital, Park Ridge, IL

### Physician Attitudes About Professionalism at Advocate Lutheran General Hospital

#### K Koo, MD; H Razzaq, MD; N Pagoria, MD; J Brom, MD; J Gravdal, MD

##### 

###### Background:

The ACGME defines professionalism as demonstrating excellence, humanism, accountability, and altruism through clinical competence, effective communication, and ethical behavior. However, no consensus has been reached on a definition and there is also difficulty generating a curriculum and learning environment that foster the development of professionalism in residents. In 2014, the Advocate Lutheran General Hospital (ALGH) Physician Commitment to Professionalism (PCP) was developed to provide specific behavioral-oriented definitions that physicians could model. The objective of this project was to examine the impact this document had on physician attitudes toward professionalism at ALGH.

###### Methods:

All attending physicians who had active appointments to the medical staff at ALGH, as well as all resident physicians and fellows who were actively enrolled in hospital-sponsored academic GME programs, received an electronic survey created through Qualtrics^TM^. The survey asked respondents if they complied with certain commitments to professionalism categorized under the broad headings of Be Safe, Be Responsive, Be Respectful, Be Professional, Be Accountable, and Be Collaborative. At three times during a 10-week period, emails with the survey link were sent, and anonymous responses were gathered.

###### Results:

Seventy-seven percent of respondents were attendings and 23% were residents; 52% were male and 48% were female. The specialties with the most respondents were internal medicine (45 participants), FM/SM (33 participants), pediatrics (29 participants), and OB/GYN (25 participants). Attending participation significantly surpassed resident participation. Gender distribution was approximately equal. We found no statistically significant difference between attending and resident awareness of the PCP. Attendings, particularly male attendings, reported exhibiting professional behaviors more often. The professional behavior most often reported was giving one's full and undivided attention to the task at hand, a behavior listed under the Be Safe heading.

###### Conclusion:

Limited participation of residents resulted in statistically significant data. Based on further analysis of survey results, comprehensive definitions of professionalism at ALGH should be constructed. It is necessary to determine factors that may be hindering the implementation of professionalism in the workplace and to determine if a different tool is needed to improve understanding and implementation of professionalism at ALGH.

**Table:** PROJECT MANAGEMENT PLAN – Tomorrow Begins Today

Vision Statement	Our vision is to increase awareness at multiple levels of the importance of faculty and resident well-being and strategies to optimize physician well-being at Advocate Lutheran General Hospital and at Advocate HealthCare.
Team Objectives	Objectives for this project were to conduct a baseline ACGME Inventory of Elements of Institutional Well-being – C-suite, program directors, residents, faculty, medical staff leadership survey the impact of our physician commitment to professionalism as a component of and marker of well-being assess hospital and system resources develop a well-being curriculum for the family medicine residency
Success Factors	The most successful part of our work was being able to make sure the conversation about physician well-being was at the forefront at multiple levels. Another successful part was quarterly Wellness Wednesdays with the family medicine residency. We were inspired by conversations about various initiatives in individual programs and beginning steps toward a more systematic approach.
Barriers	The largest barrier encountered was the transition to Epic; it consumed time, attention, and energy and was a source of burnout. Turnover and lack of support by our research department also delayed progress on our data analysis and paper preparation. Further, team members had many other often competing commitments. We worked to overcome these challenges by persevering and raising the concerns to the highest level.
Lessons Learned	The single most important piece of advice to provide another team embarking on a similar initiative is to have a clear vision, both aspirational and pragmatic, and to persevere, understanding that well-being is not an issue to be solved but to be addressed with a long-term commitment by both individuals and organizations.

## Arrowhead Regional Medical Center, Colton, CA

### Bridging Effective Communication of Patient Assignments Between Physicians and Nursing to Reduce Stressors

#### Niren Raval, DO; Teresa Smith, MBA; Rae Pierce; Greg Young, MBA; Jerome Dayao, MSN, RN, NEA-BC, CCRN; Nanette Buenavidez, MSN/ED, RN; Joanne Alexander, RN; Christina Avendano, BSBA, RN; Kedar Challakere, MD; Natalie Artinian, DO; Regina Lee, DO; Katherine Oakley, MD; Julie Smithwick, DO; Rory Smith, MD

##### 

###### Background:

A survey conducted to determine concerns that potentially affect healthcare providers' well-being showed that a prominent issue is nurses not knowing correct team assignments after patients are reassigned overnight after admission, resulting in a high volume of erroneous calls from nursing to provider teams. The purpose of this project was to analyze potential causes of stress and burnout between residents and nurses in a clinical setting and test an intervention to reduce stressors and improve well-being.

###### Methods:

Two nursing units (a test and control unit) and 2 internal medicine teams (a test and control team) were used to conduct this study. For the intervention, the admitting internal medicine test team completed team assignment index cards. These cards were given to hospital unit assistants on patient units throughout the hospital during team reassignment; the control nursing unit was excluded from this process. Hospital unit assistants updated the outside of patient charts with correct team assignments. Before and after this intervention, a survey to measure stressors was sent out via SurveyMonkey to licensed nurses on the test and control units (n=60) and physician residents on the test and control teams (n=8). The survey consisted of a modified Maslach Burnout Inventory, an abbreviated Holmes and Rahe Stress Scale, and two supplemental perception questions on the number of calls made per shift and the amount of time spent identifying the correct physician teams for patients. The abbreviated Maslach Burnout Inventory also included three additional questions on satisfaction with medicine as a career choice.

###### Results:

We found no statistically significant results from the survey responses but several promising trends. Among residents, the scores for the three subsets of the Maslach Burnout Inventory—emotional exhaustion, personal accomplishment, and depersonalization—were similar between the control and test teams. Results from the 16 residents who completed the pre- and post-surveys showed a positive trend in the satisfaction with medicine scores; the test team improved slightly while the control team experienced a decline. Results from the 35 nurses who completed the presurvey and the 24 nurses who completed the postsurvey showed a positive trend in personal accomplishment and satisfaction with medicine scores; the test unit improved slightly while the control unit experienced a decline.

###### Conclusion:

Although the study detected positive trends in 3 areas, the sample size was too small and there were challenges with subject participation and timely survey completion. We found no statistical significance between the control and test groups. Positive trends may warrant a larger pilot study to ascertain if the intervention is effective and sustainable.

**Table:** PROJECT MANAGEMENT PLAN – Bridging Effective Communication of Patient Assignments Between Physicians and Nursing to Reduce Stressors

Vision Statement	Our vision is to study the effects of systematic controls and flexibility surrounding transition-of-care communications in our hospital setting and their effects on the well-being and burnout of resident physicians and nurses.
Team Objectives	We aimed to study and analyze potential causes of stress and burnout between residents and nurses in a clinical setting; design a research study to test an intervention to reduce stressors and improve measurable well-being; and conduct the study, analyze results, and publish findings.
Success Factors	The most successful part of our work was the collaboration between physicians and nurses throughout our project. The team was inspired by the shared goal of wanting to provide the best patient care as a team.
Barriers	The largest barrier encountered was the lack of voluntary participation in the study. Approximately half of the nurses opted out of participating in the survey. Some nurses opted out before receiving the presurvey, while others expressed concerns specific to the survey questions and opted out after receiving it. It was also difficult to get both physicians and nurses to complete the surveys in a timely manner. We were able to overcome some of the concerns by having discussions with alarmed participants, and in some cases, we were able to alleviate their concerns. We overcame the slow response rate by having consistent and timely follow-up with the participants.
Lessons Learned	The single most important piece of advice to provide another team embarking on a similar initiative is to consider doing the intervention with the entire service and all nursing units instead of a couple of the teams so the sample size is adequate. We had concerns about our physician sample size being too small, but we hoped to have a much larger sample from nursing. When half of the nurses on our control and test units opted out, our sample size was too small.

## Ascension Providence Rochester Hospital (Crittenton Hospital Medical Center), Rochester Hills, MI

### Institutional and Resident-Led Wellness Interventions

#### R Brent Stansfield; Rose Natheer; Tess McCready; Sherryl Wissman; Danielle Fabry; Lucinda Wenzlick; Jacob Salman; Firas Ido; Vera Pochtarev; Tsveti Markova

##### 

###### Background:

Research shows that 25%-35% of medical residents experience burnout and that empowering physicians to participate in their own wellness initiatives can be effective. Focusing on wellness activities in addition to burnout prevention is important. We aimed to implement and assess a series of wellness interventions that involved residents in their design and instantiation. We collected quantitative and qualitative data to measure impact.

###### Methods:

This study tested if institution-led interventions, program-led interventions, resident-led interventions, and the implementation of an Annual Professional Development Symposium had any effect on wellness. A Resident Wellness Scale was developed and used to collect data at two time points. The Resident Wellness Scale subscores measured were meaningful work/ability, life security, institutional support, and social support/personal growth. A Resident Wellness Semi-Structured Interview was developed so that a third party from the GME office could conduct 15-minute interviews. The GMEC and monthly CLER Council meetings provided periodic monitoring.

###### Results:

Data from the Resident Wellness Scale showed that for meaningful work/ability, the lowest quartile became more well each PGY. However, we saw a slight decrease in wellness during the intervention period. Meaningful work scores did not improve. Overall life security was highest in PGY1s and then declined. PGY2s had the lowest life security. Institutional support was variable but highest in PGY3s. Social support/personal growth showed increases for PGY2s and PGY3s. PGY1s started with high social support/personal growth. Preliminary results from the Resident Wellness Semi-Structured Interviews identified social themes and life security themes.

###### Conclusion:

This study showed that wellness is mostly stable over time; interventions were associated with small gains in social support and life security. Program-level interventions that involved connections between faculty and residents were powerful. Intervention anticipation and participation are also important to yield positive results. Limitations were the small sample size with 3 programs at one institution; the need for many pilot programs to be replicated; and the possibility of self-selection in assessments causing biased results. Next steps should include the continuation of program-level wellness committees and Resident Council engagement at the Annual Professional Development Symposium.

**Table:** PROJECT MANAGEMENT PLAN – Institutional and Resident-Led Wellness Interventions

Vision Statement	Our vision is a sustainable culture of wellness driven by engaged, empowered residents and faculty.
Team Objectives	Objectives of this study were to implement and assess a series of wellness interventions; involve residents in their design and instantiation; and collect quantitative and qualitative data to measure impact and disseminate results in the medical education literature.
Success Factors	The most successful part of our work was empowering residents to generate their own wellness initiatives and educating them about institutional wellness resources. As a result, faculty became engaged and program-level wellness committees were formed. These committees were the most effective model for sustainable wellness activities. We were inspired by resident engagement.
Barriers	The biggest barrier encountered was that the engaged residents represented a small percentage of the larger resident population; competing priorities made engagement difficult. We worked to overcome the lack of engagement by focusing on wellness as an aspect of professional development rather than as an extracurricular consideration. This change led to faculty members being more motivated to engage. Limitations to this approach are reflected in low attendance at some events.
Lessons Learned	The single most important piece of advice to provide another team embarking on a similar initiative is to understand that faculty-resident partnerships are the key to driving change. Program directors and the GME office have the authority to mandate participation, but just their authority is not enough for active engagement. Residents have the perspective and motivation to effect change but no access to resources. Faculty working with and on behalf of residents in wellness committees garner both the power of resident motivation and legitimacy in the eyes of program leadership and the institution.

## Atrium Health Carolinas Medical Center (Carolinas HealthCare System), Charlotte, NC

### Improving Health, Inspiring Resiliency, and Promoting Well-Being: Building Resources for Our Programs

#### Eric Anderson, MEd; Suzette Caudle, MD; Eric Ekwueme, MPA; Vishal Goyal, MD; Krystle Graham, MD; Mary Hall, MD; Yasemin Moore, MHA; Sydney Primis, MD; Dael Waxman, MD

##### 

###### Background:

Individual residency programs at Atrium Health Carolinas Medical Center have developed a variety of well-being initiatives; however, there is little training of faculty, mentors, and intuitive leaders around well-being and very little organized monitoring of well-being or outcomes of initiatives. Our goal was to provide central GME resources to support individual training programs' efforts, promote objective monitoring of outcomes, share best practices among programs, and establish an advisory group for future well-being initiatives.

###### Methods:

We conducted a needs assessment of individual program activities and needs to support well-being initiatives. Results showed that 75% of residency programs had some form of established mentoring activities related to resident well-being while 25% did not. Results also showed that 42% of residency programs used the ACGME well-being survey as an indicator of improvement while 58% used no tools at all. Survey results identified opportunities for improvement in faculty/mentor training, faculty/mentor support, and other areas of logistics, including keeping scheduled meeting times.

###### Results:

We offered a mentor development program, designed and led by Center for Physician Leadership and Development experts on mentoring, for faculty and peer mentors to grow mentoring skills that support resident well-being. We also established a Wellness Council consisting of 1-2 faculty members and trainees (identified through the training program) to represent their respective programs. The council met monthly to bimonthly to gather, share ideas, and assess the efficacy of various interventions and well-being experiences. In addition to the Wellness Council and mentor program, other institutional resources identified to improve resident well-being programs included a mental health resource directory, a well-being resource list, and a resident wellness clinic.

###### Conclusion:

Programs/program directors want mentor development and institutional resources for well-being initiatives, and GME wants formal monitoring of the effectiveness of these initiatives at the GME and institutional levels. There is also a desire for ongoing discussions about best practices and an advisory group to GMEC on future growth and direction of well-being activities. A barrier encountered was the lack of time for faculty, residents, and others to plan and participate in well-being activities. Obtaining access to resources needed to accomplish desired activities was also difficult. Future plans include the potential to share, through presentation and possibly publication, the mentor development program and depending on its evolution, the Wellness Council.

**Table:** PROJECT MANAGEMENT PLAN – Improving Health, Inspiring Resiliency, Promoting Well-Being: Building Resources for Our Programs

Vision Statement	Our vision is to improve the health of residents while inspiring resiliency, promoting well-being, and creating a renowned clinical learning environment.
Team Objectives	Objectives for this intervention were perform a needs assessment of individual program activities and needs to support well-being initiatives offer a mentor development program for faculty and peer mentors focused on developing overall mentoring skills that support resident well-being establish quarterly monitoring of program-level well-being scores using the Mini-Z establish a Wellness Council, consisting of both faculty members and trainees, that will promote trainee and faculty well-being, make recommendations to the GME office and the GMEC regarding ongoing well-being initiatives, and assist in implementing and assessing the efficacy of various interventions and well-being experiences
Success Factors	The most successful part of our work was the implementation of the mentoring development workshop and the establishment of a Wellness Council. We were inspired by our programs and their continuous development to assist the residents.
Barriers	The largest barrier we encountered was project management. Early in the project, we should have assigned specific tasks to individuals on the team to remain on track. Delegation and designation of a project manager would have ensured more structure and tracking of the project. We worked to overcome this barrier by scheduling standing meetings with the project team to discuss different aspects of the project. An additional member of our Center for Physician Leadership and Development was added to the project team to assist with the facilitation of the survey, focus group, and faculty development. Other barriers were faculty and resident participation/engagement (largely related to lack of time and other obligations) and additional difficulties with access to resources and finances. Limitations of the size of the travel team was another barrier, and it hindered us at the collaborative meetings.
Lessons Learned	The single most important piece of advice to provide another team embarking on a similar initiative is to communicate with stakeholders multiple times throughout the initiative, not just once.

## Aurora Health Care, Milwaukee, WI

### Minimizing Burnout Through Three Resident Protected Time Approaches: Administrative, Personal Health, Connectedness

#### T Harrington, DO; J Vogelgesang, DO; V Dinh, MD; A Siddiqui; W Lehmann, MD; C deGrandville, MD; D Simpson, PhD

##### 

###### Background:

Well-being continues to be recognized as a critical issue for healthcare providers, with burnout rates as high as 63% among family physicians. Indirect patient care responsibilities (visit notes, inboxes, phone calls) have been identified as a significant contributing factor for burnout. Primary care physicians who spend on average 6 hours per week on EHR work outside normal clinical time are 3 times more likely to report burnout, and family physicians cite EHR and other paperwork as main causes of burnout. Our family medicine (FM) residents identified the lack of time to manage patient-related inboxes as a barrier to their well-being. Our aim was to design and implement a systems-based intervention that improved resident wellness and prevented burnout.

###### Methods:

Aurora Health Care's FM residency program implemented 3 types of protected/dedicated half-days per quarter to improve wellness and prevent burnout. One half-day was to promote personal health, allowing residents to attend their own nonurgent healthcare visits/appointments. Another half-day was to encourage a sense of community among residents, allowing residents to engage in recreational activities (eg, dining, golfing, hiking, board game day). The third half-day was to help residents reduce the burden of administrative tasks outside of scheduled work hours, allowing them to complete indirect patient care responsibilities (eg, phone calls, paperwork, chart completion, and QI projects). CG-CAHPS was the clinic metric used to measure patients' experience regarding test results communication and between-office-visits communication. The Mayo Well-Being Index was used to assess resident response, and an end-of-rotation evaluation collected data on the number of half-days taken during the rotation, scheduling barriers, how time was spent, and the degree to which the half-days made residents feel that things were more under their control. A Resident Wellness Survey was also administered that included 7 Likert items adapted from existing surveys: ability to utilize EHR, balance between education and clinical demands, feeling overwhelmed, professional growth, coworker support, meaningful work, and time spent on well-being.

###### Results:

CG-CAHPS results from June 2017 vs December 2018 showed a 5-point increase in FCC and a 4-point increase in FPC for between-visit communication and a 5-point increase in FCC and a 7-point increase in FPC for test results communication. Between February and December 2018, Mayo Well Being Index scores decreased from 3.4 to 2.2 (a score ≥5 suggests burnout). Results from the Resident Wellness Survey showed a 13% difference in responses from residents reporting that they spent an adequate amount of time on personal well-being (30% in 2018, 43% in 2019).

###### Conclusion:

Protected time for personal health, community, and administrative tasks improves residents' sense of control, well-being, and patient quality care scores. Limitations include data being limited to 12-18 months, so no long-term data are available. Next steps should include continued protected time as a built-in curriculum intervention and the continued measurement of residents' perception of well-being compared to national norms, with adjustments made accordingly. Steps should also be implemented to improve resident efficiency in administrative tasks.

## Aurora Health Care, Milwaukee, WI

### GME-Wide and Program-Specific Initiatives to Strengthen a Culture of Well-Being

#### Jacob Bidwell, MD; Tricia La Fratta, MBA; Nicole Eull, PsyD; Deborah Simpson, PhD; Timothy Lineberry, MD; GMEC Program Directors and Resident Council Representatives

##### 

###### Background:

Physician burnout is a safety, quality, and workforce issue. GME leaders convened a GMEC retreat to develop a well-being strategic plan with key system leaders. Aims for this project were to (1) serve as well-being system leaders through the development of clear GME protocols and procedures; (2) identify and provide GME-specific and systemwide resources/support to team members; and (3) improve resident and faculty well-being through residency/fellowship program-specific initiatives.

###### Methods:

Prior to the retreat, each residency and fellowship program completed the Inventory of Elements of Your Program's Well-Being Plan, and the GME office completed the Inventory of Elements of Your Institutional Well-Being Plan. At the retreat, each program director and the DIO presented key findings from their inventory and an action plan. The following focus areas for program-specific, GME-specific, and systemwide initiatives were selected: workload and job demands, efficiency and resources, social support and community at work, and work-life integration. The Mayo Well-Being Index (WBI) and ACGME well-being survey were used to measure impact.

###### Results:

Program-specific initiatives included restructuring OB/GYN weekends and overnights; providing resource half-days for family medicine; redesigning the radiology journal club/lectures; and instituting internal medicine wellness challenges. GME-specific initiatives included revising faculty contracts to reflect education roles; appointing a well-being director; providing access to confidential behavioral health services; adding a well-being question to the end-of-rotation evaluations; providing quarterly half-days for well-being purposes and access to exercise. Systemwide initiatives included adjusting contracts to align with medical group policies; partnering with system leaders; and implementing the WBI. On the WBI, the percentage of at-risk scores decreased from 17.3% to 12.7% over 11 months.

###### Conclusion:

Program-level interventions are critical and complement GME systemwide efforts. Strategies to consider going forward are monitoring Mayo WBI resources usage, unifying well-being resources with action plans via the Academic Affairs Director for Well-Being, and reviewing annual performance evaluation and well-being data.

**Table:** PROJECT MANAGEMENT PLAN – GME-Wide and Program-Specific Initiatives to Strengthen a Culture of Well-Being

Vision Statement	Aurora Health Care's GME programs will be nationally recognized for preparing our current and future physicians to help people live well—our patients, each other, and ourselves.
Team Objectives	Our GME aims/objectives were as follows: Serve as well-being system leaders through the development of clear GME protocols and procedures Identify and provide GME-specific and systemwide resources/support to team members Improve resident and faculty well-being through residency/fellowship program-specific initiatives The objectives/aims of each of the 4 participating programs were as follows: **Internal Medicine:** (1) Create a personal team for incoming residents to help with the transition into residency and (2) provide and promote education about the importance of personal health including exercise/diet **OB/GYN:** (1) Implement workload changes and time for wellness and (2) identify existing data sets and/or develop a quick check-in survey as process and outcome measures for resident/faculty well-being **Family Medicine:** (1) Design and implement a systems-based intervention that improves resident wellness and prevents burnout **Radiology:** (1) Promote well-being self-awareness, (2) identify program-specific contributing factors of burnout, and (3) implement departmental changes to improve well-being
Success Factors	Our intentional design approach as outlined in our initial application was successful. We created win-wins to reduce checkbox burnout; we used the IHI Model for Improvement (eg, aim, measures, 2 PDSA cycles, disseminate, and sustain) to meet ACGME requirements; we met requirements for quality improvement and scholarly activity (for residents and faculty); and our well-being scores are good. Working with our health system leaders, we continue to strive to make them better. We were inspired by the creativity, commitment, innovation, persistence and successes of our residency program team leaders and our GME successes in working with the system to accept clinician well-being as a crucial element of high-quality, safe patient care.
Barriers	The largest barrier we encountered was acceptance that well-being is vital to phenomenal patient care with actions/resources. We aren't done and probably never will be. Thus, we see this as a journey where we must continue to work on the recognition that well-being is vital for our common goals of amazing health for patients, providers, and populations. We must use multiple strategies; the addition of a Director for Well-Being for Academic Affairs will expand our reach and possibilities.
Lessons Learned	The single most important piece of advice to provide another team embarking on a similar initiative is that over time, a series of small steps builds recognition of the problem and identification/implementation of approaches at the system and individual levels: persistence, teamwork, data, and dissemination/spread.

## Aurora Health Care, Milwaukee, WI

### Running and Rapping our Way to Wellness: Internal Medicine Residency Approach to Preventing Burnout

#### Siri Neelati, MD; Kathy Scigacz, MD; Prakash Nallani, MD; Richard Battiola, MD; Tanya Shah, MD; Xiao Xiao Qian, MD; Deborah Simpson, PhD

##### 

###### Background:

Between 22%-60% of practicing physicians are reported to have experienced burnout that stems from lack of work satisfaction, overwhelming schedules, and loss of support from colleagues. Internal medicine (IM) ranks among the highest of all specialties in burnout, with rates to 76%. Residents recover from existential burnout by feeling validated, forming connections with patients/colleagues, and increasing competence by engaging in career development initiatives. Medical students whose aerobic exercise and/or strength training habits are consistent with CDC guidelines appear less likely to experience burnout, and they appear to have a higher quality of life. The goal of this intervention was to promote exercise and relationships with colleagues by (1) creating a personal team for incoming residents to help with the transition into residency and (2) promoting the importance of personal health. Long-term goals included (1) monitoring the effectiveness of interventions during the academic year and (2) gaining a better understanding of the ongoing trends that contribute to resident burnout.

###### Methods:

A Resident Advising Program for Success (RAPS) team that included a faculty advisor, a senior resident, and a junior resident was formed for internal medicine. Incoming interns were assigned to a PGY2/3 resident in continuity clinic and a corresponding RAPS team. Interns were welcomed via an email from their assigned RAPS team that included a team photo and fun facts about each member. The RAPS team was to conduct quarterly check-ins with the interns and engage in 3 out-of-hospital bonding activities. A month-long wellness challenge was also implemented and promoted in the internal medicine newsletter; residents were challenged to follow AHA guidelines by engaging in physical activity 4 days per week and healthy eating 3 days per week. Suggested applications and resources were also published in the newsletter to educate and encourage participation in the challenge. A weekly 3-item survey was sent via MedHub per AHA guidelines asking (1) the number of days in the last week that the intern engaged in >30 minutes of moderate and/or 25 minutes of vigorous intensity exercise, (2) if exercise was paired with other activities/priorities in their lives, and (3) a rating of the intern's overall health (physical, emotional) in the past week. Semistructured 3- to 5-minute individual interviews with interns were also conducted to gauge their opinion of the overall value of RAPS.

###### Results:

In June 2018, 11 interns attended RAPS events, and 4 attended in January 2019. A total of 13 residents were interviewed, and 85% (11) welcomed the idea of a more structured peer advisor program, while 15% (2) felt that the program was not necessarily helpful but had potential. Participation at weekly meetings with peer advisors at the clinic was 100%, but participation outside of the clinic setting was 0%. The number of interns who worked out 4-7 days per week increased from 10% at baseline (prior to the fitness challenge) to 17% during the challenge, and then declined to 14% after the challenge. Mayo Well-Being Index scores for IM residents increased from 1.9 (February 2018) to 2.7 (December 2018), but the mean score was well below the score of ≥5 that predicts resident burnout and associated symptoms such as low mental quality of life, high fatigue, or recent suicidal ideation.

###### Conclusion:

Initiating core teams before residency begins may be helpful in the initial transition and could lead to long-term trusted relationships. Simplicity is key, and frequent reminders yielded higher completion rates but were cumbersome for the team. Barriers included limited data and the possibility that attaining data with surveys may increase burnout by adding more to be done. Next steps should include the formation of a Residency Program Wellness Committee to continue to sustain and build interventions.

## Aurora Health Care, Aurora St. Luke's Medical Center, Milwaukee, WI

### Efficacy of Well-Being Self-Awareness and Departmental Interventions Within a Single Radiology Residency Program

#### Mason Brown, MD; S Reimer, MD; N Patel, MD; N Dickson, DO; W MacDonald, MD

##### 

###### Background:

A Medscape survey with >15,000 physician responses from 29 specialties showed that radiology ranks 7th among the top 10 burnout specialties, with a burnout rate of 45%. The goals of this project were to promote well-being self-awareness in the Department of Radiology, identify program-specific contributing factors of burnout, and implement departmental changes to improve well-being.

###### Methods:

Interventions included an after-hours journal club at a local restaurant, a new resident welcome party hosted by faculty, and a weekly core radiology lecture series that focused on team-based exercises and resident camaraderie. Measures used to assess impact were the Mayo Well-Being Index and a Work Relationships and Job Satisfaction Survey.

###### Results:

The highest rated initiative was the new resident welcome party hosted by faculty. Resident burnout, being overwhelmed, anxiety/irritation, emotional hardening, daytime sleepiness, and compromised health all demonstrated a decrease in monthly frequency after the interventions. Job satisfaction and personal/family time remained unchanged. The sense of resident-to-resident connectivity and resident-to-faculty connectivity increased. The most highly rated activity from a list of proposed future initiatives was a resident vs faculty annual sporting matchup. The Mayo Well-Being Index was not perceived as a helpful awareness or assessment tool.

###### Conclusion:

Overall resident well-being improved over the course of 2 academic years while under surveillance and with 3 program interventions. Increased time with friends and family was perceived to offer the most significant improvement in well-being. Next steps are to assess interest for an annual sporting matchup between resident and faculty teams.

## Aurora Health Care, Milwaukee, WI

### OB/GYN Resident Well-Being Focused on Workload and Wellness Time: Measured Using a 3-Item Well-Being Check-In Card

#### Naomi Light, MD; Morgan Altinok, DO; Carla Kelly, DO, MMM; Deborah Simpson, PhD

##### 

###### Background:

Between 22%-60% of practicing physicians are reported to have experienced burnout, but OB/GYN resident burnout has been reported at 90%. Contributors to burnout include workload and job demands, control and flexibility, poor work-life integration, and checkbox requirements (filling out surveys, module requirements, duplicates, paperwork). Our resident well-being intervention aims were to implement workload changes and time for wellness and to identify existing data sets and/or develop a quick check-in survey to use as process and outcome measures for resident/faculty well-being.

###### Methods:

In July 2017, three workload protocols were changed: (1) for weekend rounding protocols, residents continue to round on all antepartum and gyn patients at the end of each 24-hour shift, but now faculty complete all postpartum rounding; (2) weekday postpartum rounding was redistributed, decreasing the number of patients per junior resident from >10 patients to a maximum number of 6-7 patients per resident; and (3) residents have no service obligations on Sundays, and we have two months of no residents on night float. In addition, quarterly wellness mornings began in September 2017, using protected education time for faculty and resident physicians. Measures used to evaluate the impact of the interventions were well-being check-in cards, the Press Ganey Engagement Survey, and the Mayo Well-Being Index.

###### Results:

Six well-being check-in cards were completed in September 2017-December 2018. Overall, the amount of time spent on personal well-being increased from baseline in September, and the number of residents who identified their work as meaningful declined at some timepoints, but at several timepoints improved over baseline. The Mayo Well-Being Index decreased from 3.2 to 2.9 over 6 months, but increased by 1.0 in December to 3.8.

###### Conclusion:

The Mayo Well-Being Index provides a benchmark with national comparisons for OB/GYN residents, and the findings appear to equal those for the well-being check-in cards. Strategies going forward are to use protected time for data collection, continue to implement interventions, and add/adjust as needed.

## Bassett Medical Center, Cooperstown, NY

### ResWell: Design and Implementation of a Resident Wellness Program

#### Kristin Baker; Connor Davenport; Natalia Golub; Ethan Talbot; Sara Albright; Melissa Hochbrueckner; Melissa Scribani; Jossy John; Caroline Gomez-Di Cesare; James Dalton

##### 

###### Background:

Nationally and locally, burnout among healthcare workers is increasingly prevalent, negatively impacting all elements of healthcare. Surveys of our own resident population reflected that our trainees are not immune and are at risk for burnout. In response, a resident-led group designed, implemented, and is evaluating and augmenting a resident wellness program (ResWell) at Bassett Medical Center. The aim of this project was to improve well-being and decrease burnout among all Bassett residents (internal medicine, general surgery, and transitional-year trainees).

###### Methods:

A Resident Wellness Committee of new and returning residents was formed in June 2017; they met regularly and collaborated with institutional well-being leaders and advocates to create ResWell. The program was designed to engage residents in trainee-directed activities and interventions to increase the level of well-being among the resident community. The program included various interventions focused on growing professionally (eg, dinner workshops); improving physical health and nutrition (eg, Zumba workshops); improving mental health and reducing stress (eg, Arts in Healthcare); and improving community building and communication (eg, potlucks). A survey of resident wellness was administered 3 times through the year that combined an expanded Resident Well-Being Index, the Brief Resiliency Scale, open questions regarding program culture and specific activities, medical error questions derived from the Stanford Professional Fulfillment Index, and open-ended questions regarding the psychological effects of errors and help-seeking (added in October 2018). Brief postactivity surveys solicited feedback on the activity.

###### Results:

Neither the Resident Well-Being Index score or the Brief Resiliency Scale changed significantly during the study period. However, residents were engaged in the wellness activities, feedback was generally positive, and activities were considered meaningful. Resident-led activities resulted in a collaboration between ResWell, Nursing, and Pastoral Care, resulting in initiation of the Arts in Healthcare program that is now available to employees across the system. Responses to the questions added in October 2018 provided insights into how residents manage errors emotionally and residents' help-seeking behaviors. These results helped strengthen resident mental health services and make them more visible.

###### Conclusion:

The well-being needle can move using an organized multifaceted approach and a small investment of resources. Designing and implementing resident-directed activities and responding to constructive and instructive feedback enhance resident participation in activities. Emotional and administrative support to the resident members of ResWell is paramount for the group to be sustainable and to prevent burnout and attrition of those actively trying to prevent burnout. Limitations included time conflicts and restrictions among residents that created scheduling challenges and increased stress upon those working to improve well-being. It was also a small program, and few residents responded to surveys, incumbering the interpretation of quantitative data. Next steps should include increasing administrative support to promote sustainability, reevaluating ways to assess ResWell's impact on resident well-being, and conducting an ongoing reassessment of interventions and activities to identify gaps in achieving the vision and mission of ResWell.

**Table:** PROJECT MANAGEMENT PLAN – ResWell: Design and Implementation of a Resident Wellness Program

Vision Statement	Our vision is for resident physicians at Bassett Medical Center to be intellectually, emotionally, spiritually, and physically prepared to engage in a stimulating and fulfilling life and career. The mission for ResWell is to engage our residents in trainee-directed activities and interventions to elevate them toward a higher level of well-being.
Team Objectives	Objectives for this project were to make ResWell a central player in the development of wellness activities and interventions for the residents at Bassett Medical Center plan and organize activities that address each domain of well-being have the institutional GME office include residents in decisions and strategies that pertain to their wellness have ResWell members meet with the CLER site visitors when they are at Bassett have the steering committee supervise and administer the wellness and resiliency survey three times annually observe participation in ResWell activities and obtain feedback receive administrative and financial support from the GME office
Success Factors	The most successful part of our work was the residents' recognition that they are valued by the institution and that they have some control over their lives. Residents were engaged with wellness activities and found value in the activities. Resident mental health services are strengthening and becoming more visible, and residents are more aware of resources. We are inspired by spinoff programs and interventions, such as the Arts in Healthcare program.
Barriers	The largest barrier encountered was attrition of resident participation in the ResWell steering committee. We worked to overcome this challenge by providing the committee with more consistent administrative support. We received limited responses to anonymous periodic surveys. We worked to overcome this challenge by providing multiple mechanisms to access the surveys, utilizing ResWell committee members to encourage participation and publicizing the survey results along with changes that occurred because of the results.
Lessons Learned	Providing emotional and administrative support to the residents who lead ResWell is paramount to make the group sustainable and to prevent burnout of those actively trying to prevent burnout. Resident participation in activities is enhanced by making the activity resident directed and by encouraging and responding to constructive feedback.

## Baylor University Medical Center at Dallas, Dallas, TX

### Resident Wellness Week

#### Cristie Columbus, MD; Jennifer Olvera, MBA; Natalie Gittus, JD; Tom Cox, PsyD; Jennifer Jolly; Kaki Whitty, MD; Julia Berry, MD

##### 

###### Background:

The years that trainees spend in medical school, residency, and fellowship are formative in the development of a physician's long-term identity and habits (both healthy and unhealthy) for managing the stressors of the modern healthcare environment. The critical time to ensure that physicians are developing healthy habits of self-care and stress reduction occurs almost exclusively during training. The goal of this project was to implement an annual wellness week to reintroduce trainees to a wide variety of strategies to combat burnout and improve wellness during training.

###### Methods:

We initiated an annual wellness week in April 2018; trainees were exposed to multiple resources that decrease stress and improve well-being. Activities included yoga, pet therapy, massages, creative art activities, and completing a self-care wheel. We also provided educational handouts on topics such as healthy snacks and meals available in the hospital, sleep hygiene, mindfulness and meditation, financial wellness, and tips for managing stress. When we began this project, a measurement tool wasn't in place, and no baseline data could be collected. We used the American Medical Association's Mini-Z Resident Physician Burnout Survey to obtain our first measurement of wellness in September 2018. The timing of the survey was strategic, as we wanted to capture the wellness of all trainees but were particularly interested in the wellness levels of first-year residents and fellows entering our institution. We will give the survey again to graduates of our programs in spring 2019. Our ultimate goal is for trainees to graduate from our programs as mentally healthy or healthier than when they started training. To measure this, we will continue to survey trainees right after they have started training and again right before they graduate.

###### Results:

One-hundred seventy-three trainees completed the survey (65% of our trainees). Ninety-six percent of trainees were satisfied with our workplace, 0% were unsatisfied, and 4% were neutral. Thirty-four percent described themselves as having no symptoms of burnout, 54% felt at least some levels of stress, and 13% qualified as burned out. The combined result of these two metrics gave us a Mini-Z score of 78.5%. Eighty percent is the target for zero burnout and a joyful workplace. Trainees reported a desire to continue to engage in similar activities on their own and requested that the institution host monthly wellness breaks in addition to a second wellness week for year-round reminders. Positive feedback from trainees led us to implement monthly wellness events in the 2018-2019 academic year (such a**s** visits with the therapy pets, healthy snack breaks, holiday themed activities, and evening socials) and a second annual wellness week in April 2019.

###### Conclusion:

In post-wellness week surveys, trainees reported that it was helpful to be reminded of simple ways to decrease stress like taking a short walk or a snack break. During wellness week, trainees were able to reconnect with some activities that they enjoy but hadn't made time for in a while (eg, art, time with animals, time outside, and physical activity). A limitation was that many trainees were unable to participate due to night shifts, vacations, and patient care responsibilities. Another limitation was that we did not do a wellness survey prior to wellness week, so we are unable to assess its impact on overall trainee health, but we are hopeful that the positive results on our first wellness survey were, in part, due to the wellness week project.

**Table:** PROJECT MANAGEMENT PLAN – Resident Wellness Week

Vision Statement	Our vision is to develop long-term and innovative strategies to improve resident well-being across a variety of areas including community, mental health, exercise, healthy eating, good sleep habits, and work-life balance. Ideally, we would like to see healthy habits form during training that will continue to aid our graduates in maintaining wellness and good health throughout their careers.
Team Objectives	Our objective for this project was to provide trainees with education on tools and resources through an annual wellness week. Goals were to (1) teach trainees a wide variety of strategies to combat burnout and improve wellness and (2) ensure that trainees are able to self-identify when they are stressed, burned out, or struggling mentally/emotionally.
Success Factors	The most successful part of our work was the amount of trainee participation in various activities. We were inspired by trainee enthusiasm in the wellness events and how simple activities, like bringing in therapy dogs, were the most effective.
Barriers	A large barrier was trainee engagement in the activities, including time to participate and support from medical staff to allow participation. We worked to overcome this challenge by using house staff champions, overly communicating the schedule of activities, scheduling activities at different times of day, and including a raffle of donated prizes.
Lessons Learned	The single most important piece of advice to provide another team embarking on a similar initiative is to involve trainees in the planning.

## Baystate Health, Springfield, MA

### Walk a Day in My Shoes

#### Ryan Quarles, MD; Donald Kirton, MD; Reham Shaaban, DO; Kevin Hinchey, MD; Heather Z Sankey, MD, MEd

##### 

###### Background:

Provider burnout has been identified as a public health crisis, and efforts to study and improve wellness are being undertaken by most major academic medical centers. The relationships we build at work can make or break our work environment and make a significant difference between dreading work and enjoying it. When providers dread the work that they do and the environment they are in, having empathy for patients and coworkers is affected. Our goal was to improve empathy by building stronger relationships within the healthcare team through a shared understanding of roles and how we can work better together through shadowing experiences.

###### Methods:

Incoming residents in internal medicine (IM), pediatrics, and OB/GYN were scheduled to shadow floor nurses in their respective units. The shadowing sessions were 4 hours in length with nurses who volunteered for the experience. After each session, the residents and nurses were debriefed with a predetermined set of questions and an open discussion on observations, lessons learned, and commitments with their program leadership as well as a nurse manager.

###### Results:

Twenty-two incoming IM and pediatrics residents shadowed floor nurses. Residents in OB/GYN were scheduled for shadowing with volunteer nurses, but a misunderstanding by staff caused the residents to shadow senior residents instead. Key findings from this project were (1) resident physicians are unaware of the level of involvement and expertise nurses have in the care of their patients and (2) experiencing the workflow and demands of a nurse's day creates a deeper understanding and appreciation for their role on the team. Common themes identified were nursing role on the healthcare team; appreciation of nursing clinical knowledge and skills; workload of nursing staff; and nurses as a source of learning, communication, and safety.

###### Conclusion:

Project barriers included the lack of recognition across the department of the value of shadowing; building this experience into protected orientation time is very important. Next steps will include reworking the debrief sessions to have nurses and residents debrief separately; completing a preexperience interview to understand preexperience knowledge; and reassessing the practicality of surveying all residents on attitude.

**Table:** PROJECT MANAGEMENT PLAN – Walk a Day in My Shoes

Vision Statement	Our vision is to improve empathy by building stronger relationships within the healthcare team through a shared understanding of our roles and how we can work better together through shadowing experiences.
Team Objectives	Objectives for this project were to assess the short- and long-term impact on empathy, understanding, and communications of existing nurse-resident shadowing experiences during orientation for our programs in internal medicine and OB/GYN expand the program to other residencies and programs to have residents shadowing each other across specialties expand the shadowing experience to other healthcare students and residencies and even wider into the system as part of the systemwide provider orientation develop a program by which nurses can shadow residents and attendings to understand their workflow and structure
Success Factors	The most successful part of our work was the organic learning that occurred when the residents observed that many of their assumptions were incorrect and their ability to build a better understanding of the nurses' role and how to work with them. We were inspired by the quality and quantity of reflection by the residents and how open the nurses and residents were to participate.
Barriers	The largest barrier encountered was the recognition of the value of shadowing across the department. We worked to overcome this challenge by ensuring that the whole department was aware of our intentions and goals with this activity and by building nurse-resident shadowing as a protected activity for more residents going into the next orientation period.
Lessons Learned	The single most important piece of advice to provide another team embarking on a similar initiative is to build this experience into protected orientation time.

## Billings Clinic, Billings, MT

### Studies on Physician Resiliency and Well-Being in Rural Montana

#### James Jackson, MD; Kylie Ebner, DO; Robert Renjel, MBBS, JD; Virginia Mohl, MD, PhD; Ashley Dennis, PhD; Keith Davis, MD; Sarah Peila, MD; Joseph Peila, MD; Mark Lee, MD, FACP

##### 

###### Background:

The mission of the Billings Clinic internal medicine (IM) residency program is to train expert physicians to care for complex medically ill patients in rural environments. Successful recruitment and retention of physicians in these rural communities requires improved understanding of resilience and well-being to promote joy in practice.

###### Methods:

Two resident physician-led projects were funded to improve our understanding of physician resilience and well-being. The aim of the first project, Decreasing Burnout in Medical Residency: Implementing a Balance Coaching Program, was to examine whether IM residents who participate in a program designed to improve resident coping and communication experience an improvement in their well-being scores and a decline in their burnout scores. The second project, Qualitative Analysis of Internal Medicine Physician Recruitment and Retention in Rural Montana and Northern Wyoming, aimed to examine the common factors that impact resiliency and well-being among IM physicians practicing in rural Montana and Wyoming. All first-, second-, and third-year IM residents working at Billings Clinic were invited to participate in the residency burnout project. Participants (n=17) took baseline measurement surveys (a demographics survey, the Professional Quality of Life 5 [ProQOL 5], and the Mental Health Inventory). Participants attended balance group sessions. Follow-up surveys (ProQOL 5 and the Mental Health Inventory) were done at 4 and 8 months. For the physician recruitment and retention project, the Montana Medical Association database identified rural (areas with a population <50K) internists and IM specialists, and we also obtained peer referrals (n=63). Data were gathered through semistructured interviews, a 6-question survey, demographics, and 1:1 recorded/transcribed interviews. We stopped gathering data at saturation (n=17, target∼25). Data were analyzed by coding transcripts, thematic analysis, and checking results with the interview data source.

###### Results:

Results from the ProQOL 5 showed a decrease in mean resident burnout scores at 4 months compared to baseline, moving from a medium to a low score. Results from the Mental Health Inventory demonstrated an increase in resident mean mental health scores at 4 months compared to baseline. No correlation was shown between outcome measures and balance group attendance. Participants in the physician recruitment and retention project had an average age of 48.4 (SD=10.8 years); 76.5% were male and 23.5% were female; 100% were from the United States, 94.1% were MDs, 5.9% were DOs; 23% had a rural upbringing; 94.1% were married; and 88% had more than 2 children. Recruitment factors included having friends or family in the region, lifestyle preference, upbringing, scope of practice, the opportunity to get to know patients, and a good work/life balance. Results suggest that retention factors include good relationships with administration, the flexibility to shape practice, scope of practice, and the ability to get to know patients. Factors making participants want to leave the practice include isolation and too much administrative work.

###### Conclusion:

Residents at Billings Clinic experienced a medium level of burnout at project onset. No correlation was shown between outcome measures and balance group attendance, but qualitative data suggest that residents who attended balance groups enjoyed the opportunity for confidential, small-group discussions with their peers. Barriers to the residency burnout project include scheduling conflicts, occasional perceived dissonance between balance group session topics and relevancy, mixed feelings about group/peer interactions, and some residents' uncertainty of the intervention's benefits. Limitations were the small sample size, inconsistent attendance and survey completion, inability to adequately pair data, and selection bias. For the recruitment and retention project, data gathering barriers included challenges in connecting with interviewees, administrative office staff barriers, scheduling conflicts with busy practices, the tendency to request face-to-face interviews, and a lack of interest to be involved in a resident study. Geographic barriers included the paucity of rural airport access and automobile commute times being more than 5 hours each way.

**Table:** PROJECT MANAGEMENT PLAN – Studies on Physician Resiliency and Well-Being in Rural Montana

Vision Statement	Our vision is to be a learning laboratory for the rural healthcare workforce of the future and for our graduates to be healthy and balanced experts in the care of complex medically ill patients in rural environments.
Team Objectives	Our objectives were to address challenges faced by residents to support resilience and well-being and to survey internal medicine physicians in rural areas to examine factors associated with professional satisfaction in rural practice.
Success Factors	The most successful part of our work was collaborating across our organization on physician engagement and well-being and creating resident leaders and faculty mentors. We were inspired by the opportunity to collaborate with other institutions and by our residents' engagement.
Barriers	The largest barrier encountered was the transition to a new program director in the middle of this project. We worked to overcome this by working with our Office of Medical Education.
Lessons Learned	The single most important piece of advice to provide another team embarking on a similar is to choose stakeholders thoughtfully.

## Cedars-Sinai Medical Center, Los Angeles, CA

### Improving Housestaff Access to Wellness Resources

#### Mark Noah, MD; Betsy McGaughey, EdD, MS; Jeffrey McKelvey, BA; Marc Cuenco, BA

##### 

###### Background:

Cedars-Sinai provides extensive well-being resources to housestaff, but input from housestaff indicated a lack of awareness of many existing resources and the restriction of having to research and access the resources from a campus-based computer. The focus of this project was to enhance access to the well-being resources by providing housestaff with a curated compilation of local and national wellness resources that they could access via their mobile devices.

###### Methods:

The Box file sharing system was selected as the most feasible option to manage and share wellness information. Information about and links to available resources were selected, compiled, curated, and categorized in a Box folder based on input from the Resident Wellness Committee. All housestaff were sent a personalized email invitation to join the Box folder. New housestaff orientations between June and August 2018 included a presentation on downloading the Box app and using/accessing the materials on attendees' mobile devices; attendees were also offered in-person technical support. All continuing housestaff were provided the presentation on downloading the app, and rates of invitation acceptance to join the Box folder were monitored. Housestaff were surveyed approximately 10 months after the Box file resource became available to assess perceptions of usefulness and effectiveness of the information and to elicit their personal level of burnout using a single validated question on the survey.

###### Results:

Eighty-eight percent of housestaff (513 of 581 of new, continuing, and terminating) accepted the invitation to access the Box folder. After excluding the 145 terminating housestaff, the remaining 436 individuals were divided into new housestaff (n=163) and continuing housestaff (n=273). Both groups received 5 survey questions; the continuing group was asked an additional question about their awareness of the wellness resources prior to the app. The overall response rate was 53%. Survey results showed that 81% of the new group downloaded the app to their smart phones, and 47% of continuing housestaff downloaded the app. Among those who downloaded the app, 37% felt that their well-being improved with the resources provided, 19% disagreed, and 44% had no opinion. Six percent felt that the resources section on feeling down or anxious was useful. More than 60% of respondents reported not feeling burned out, 27% had two symptoms of burnout, and 9% had severe symptoms of burnout. Both groups ranked the same four of the eight categories of wellness resources most useful: benefits, health and fitness, discounts, and recreation in Los Angeles.

###### Conclusion:

Providing a setting where housestaff were directly shown how to download the resources on their smart phones was more effective than reminding them to download the app through multiple emails and program director encouragement. More than one-third of residents felt that wellness resources that can be accessed through their smart phones can improve their overall well-being, but results showed that downloading the wellness app did not have an impact on the level of burnout felt by trainees. A limitation was that measurements did not have adequate power. Next steps include continuing to monitor survey results to determine changes over time. We will also continue the work of the subcommittee and collaborate with similar housestaff forums and medical staff initiatives.

**Table:** PROJECT MANAGEMENT PLAN – Improving Housestaff Access to Wellness Resources

Vision Statement	Our vision is to promote resident well-being by creating an environment that fosters resiliency, a positive work-life balance, and a supportive community.
Team Objectives	Our objective was to consolidate access to resources and enable them to be accessible in a resident-friendly mode through a mobile application.
Success Factors	The most successful part of our work was developing an enthusiastic multidisciplinary group that has engaged in providing well-being activities for housestaff beyond this project. We were inspired by everyone's sustained interest in promoting resident well-being.
Barriers	The largest barrier encountered was getting good data to judge the efficacy of the project. We administered the Resident Wellness Scale multiple times but found that the scale did not fit with the evaluation of our project, so we needed to come up with another data collection plan.
Lessons Learned	The single most important piece of advice to provide another team embarking on a similar initiative is to plan usable data collection.

## Christiana Care Health System, Newark, DE

### A Comprehensive Systems Approach to Resident Well-Being

#### Vanessa Downing, PhD; Mark Mason, PhD; Heather Farley, MD, MHCDS, FACEP; Greta Ehrhart, MPA; Sam Van Horne, PhD; Brian Levine, MD; Lisa Maxwell, MD; Margaret Kennan, PhD; John Donnelly, MD; Loretta Consiglio-Ward, MSN; Christina Edwards; Melissa Cummings, MD; Elizabeth Shy, MD; Heather Bari-Brown, MD

##### 

###### Background:

Christiana Care Health System (CCHS) provides a clinical learning environment for more than 280 residents/fellows from more than 31 residency and fellowship programs. Research has found deficits in well-being and high incidences of burnout among residents and early career physicians. In 2016, CCHS founded the Center for Provider Wellbeing (CPW) to foster joy and meaning in work for providers and their teams. Through a partnership with CPW and Academic Affairs, our NI VI team aimed to address resident burnout rates via a proactive, comprehensive approach. The goal of this project was to go beyond simple assessment of burnout to gain a better understanding of the drivers of engagement/fulfillment and burnout across numerous residency programs and to begin a process of meaningful organizational and system change.

###### Methods:

The CCHS well-being strategic plan had 4 focus areas: well-being education (quarterly well-being sessions, annual intern well-being intensives, Balint groups for residents, reflective writing workshops, and career development panels), efficiency of practice (biannual resident focus groups and bimonthly GMEC wellness presentations), response to resident crises (resource liaison line, EAP for emergency and mental health resources, peer support program, and provider litigation program), and culture of well-being (annual performance evaluation wellness goals for residency programs, resident well-being committees, resident council representative, psychological/learning assessment services, resident retreats, and Residents' Day). Measures used to assess outcomes of the implementations were the Annual Provider Wellbeing Survey, participation in resident/fellow focus groups, use of the resource liaison line, and annual performance evaluation well-being goals.

###### Results:

Among residents who answered the Annual Provider Wellbeing Survey in both years, declines were seen in professional fulfillment and control. Participation in quarterly focus groups grew from 2 programs in 2017, to 10 programs in 2018, to 14 programs in 2019. The number of resource liaison calls was <5 in May 2017, increased to >10-15 calls in the October to December 2017 time frame, decreased to <5 between April and June 2017, and increased to >20 calls between January and February 2018. Annual performance evaluation goals varied in scope and focus, from Balint groups to new call room.

###### Conclusion:

Assessing the impact of culture change efforts is a long, slow process; identifying an immediate benefit will be difficult. Residents, residency leadership, Academic Affairs, and CPW faced a steep learning curve associated with co-creation of well-being goals. Next steps include quantitative data collection for 2019; making well-being goals a standard aspect of the annual performance evaluation process; implementing biannual residency program focus groups; and evaluating APE wellness goals to determine achievability, impact, and co-creation with residents/fellows.

**Table:** PROJECT MANAGEMENT PLAN – A Comprehensive Systems Approach to Resident Well-Being

Vision Statement	Our vision is to connect the value of strategic investment in provider well-being to the advancement of our institution's core values of Love and Excellence and the Quadruple Aim and to optimize the experience of providing care within our organization.
Team Objectives	We aimed to address resident well-being at the individual, programmatic, and organizational and leadership levels of the institution. Objectives were to engage residents in well-being education and programming cultivate resilience and social support through quarterly psychologist-led sessions focusing on reflection and self-awareness promote mental health and encourage help-seeking behaviors by developing institutional policies that protect clinicians' dignity, safety, and privacy, along with increasing access, familiarity, and exposure to psychologists initiate clinical learning initiate clinical learning environment change via the residency program annual performance evaluation process with Academic Affairs
Success Factors	The most successful parts of our work were the addition of well-being goals to the annual performance evaluation, a marker of significant partnership between GMEC, Academic Affairs, the Center for Provider Wellbeing (CPW), and residents/fellows increased consultation, involvement, and collaboration between program leadership and the CPW increased help-seeking behaviors by residents and fellows in distress continued expansion of quarterly well-being sessions to new residency and fellowship programs We were inspired by collaborative, mutually respectful relationships among Academic Affairs, GMEC, CPW, and residency/fellowship program leadership the openness, interest, engagement, and participation among residents and fellows in discussing topics such as burnout, wellness, and organizational culture senior leadership/CEO/C-suite buy-in and institutional support Christiana Care organizational values and shift toward Love and Excellence
Barriers	The largest barriers encountered were fostering open communication and collaboration between resident well-being champions and program leaders helping program directors feel empowered to enact real organizational change time We worked to overcome these challenges by attending and participating in monthly GMEC meetings forming collaborative work groups and teams across departments and roles promoting cooperation by framing our roles as collaborative consultants collecting data to bolster initiatives creating an FTE entirely dedicated to the well-being of those in the clinical learning environment
Lessons Learned	The single most important piece of advice to provide another team embarking on a similar initiative is to develop data-driven initiatives for resident physician wellness at both the individual and organizational levels. Individual and organizational factors are highly interrelated.

## Cleveland Clinic Akron General Medical Center, Akron, OH

### A Multipronged Approach to Creating a Culture of Resiliency Support

#### Nathan Hieb, MD; Heather Snyder, DO; Narine Sharkhatunyan, MD; Jennifer DeMarco, MD; Kristin Filipowicz, MEd; Coda Derrig, PhD; Titus Sheers, MD; Lori Smith, MBA; Elias Traboulsi, MD; Cory Chevalier, MD; Nairmeen Haller, PhD; Cheryl Goliath, PhD

##### 

###### Background:

In addition to mental health problems, burnout also has physical effects, including a significant increase in motor vehicle accidents. Studies have tested different interventions to improve resiliency with varying levels of success, but no programs currently address caregiver resilience in a timely and consistent manner. The aims of this project were to survey caregivers to assess current practices across residency programs related to caregiver wellness, determine gaps in support, and develop and implement a multipronged program to support resident and faculty resiliency.

###### Methods:

Project participants included Cleveland Clinic Akron General residents and core faculty from emergency medicine, family medicine, general surgery, internal medicine, OB/GYN, and orthopedic surgery training programs. A needs assessment was administered to determine knowledge of existing resources, gaps in support, and desired resources. Changes were implemented based on the responses to the needs assessment. Measures used to assess the impact of the changes will include responses to ACGME well-being questions 1 year following implementation (2020) of the changes to determine if a positive shift occurs in the culture of resiliency support (responses to ACGME well-being questions were used as a baseline measure of resiliency culture and support); Caring for Caregivers QR code utilization; and EAP resident utilization before and after initiative changes.

###### Results:

From the responses to the needs assessment, the following changes were implemented: (1) integrated a Caring for Caregivers slide with a QR response code in morbidity/mortality conferences for respective programs; (2) increased visibility of the EAP in all training programs through more frequent didactic presentations; (3) developed laminated Caring for Caregivers signs and posted them in resident lounges, bathroom stalls, and call quarters; and (4) created a resources intranet page.

###### Conclusion:

Eliminating the stigma associated with seeking help is the first step in changing the culture of resiliency at our institution. Fear of repercussion and lack of anonymity were key barriers to a successful culture of resiliency support. Many of the resources requested by residents and core faculty were already in place, necessitating increased visibility and awareness. Protected time and insurance coverage will be essential to sustaining the culture of resiliency support. Limitations of this project include deidentified responses; an unlikely 100% response rate for each measure; and the fact that the same residents and core faculty may not respond to or participate in any one measure, so results may not reflect a true change from baseline.

**Table:** PROJECT MANAGEMENT PLAN – A Multipronged Approach to Creating a Culture of Resiliency Support

Vision Statement	Our vision is to create a culture of resilience for our caregivers in a safe environment where they feel empowered and supported when faced with the inevitable challenges of providing world-class care for their patients.
Team Objectives	Objectives of this project were to assess current practices in each of the residency programs related to caregiver wellness, survey caregivers to determine gaps in support, and create a plan to address their needs.
Success Factors	The most successful parts of our work were our engagement with the residency programs, our response rate, and the ability to create a more visible presence for our employee assistance plan resources. We were inspired by the thoughtfulness of the resident responses to the resiliency questions.
Barriers	The largest barriers encountered were residents' availability, follow-up on team assignments, and timing of survey rollouts. We worked to overcome these barriers by increasing communication from team leadership.
Lessons Learned	The most important advice to provide another team embarking on a similar initiative is have clear messaging that explains the purpose of the project have NI VI team members who attend resident meetings inform the residents about the project vs relying on chief residents and program directors use a QR code (it made the process accessible for the residents)

## Community Health Network, Indianapolis, IN

### “We” for Wellness

#### Kathy Zoppi, DIO, PhD; Jesse Clark, DO; Stephanie Nader, LCSW; Ann Cunningham, DO; Chris Basom, DO; Joanna Edwards, MD; Blane Riley, DO; Telycia Johnson, DO; Christine Hopp, DO; Britney Roberts, DPM

##### 

###### Background:

Community Health Network is harnessing collaborative relationships with key stakeholders to implement initiatives for wellness on an institutional level. Our goals for this project were to decrease resident burnout, as measured by the Wayne State University Resident Wellness Scale (RWS), by 5% and to encourage program-specific wellness initiatives within each of our residency programs.

###### Methods:

Residents across the network were given one dedicated half-day for wellness in the fall and in the spring. On this day, they chose to engage in organized wellness activity sessions or a personal wellness activity of their choice. Residents unavailable for these standardized days (due to night float) individually selected a wellness half-day immediately following the night-float rotation. If a resident was on vacation during the half-day, they did not have to take the half-day from allotted vacation time. Residents were surveyed using the 10-item RWS and the annual ACGME survey to monitor any impact on well-being. In January 2018, the ACGME resident and faculty surveys were administered and administered again in January 2019. In July 2018, the baseline RWS was administered to all residents. The RWS was administered again in December after the intervention that was implemented between August and September of 2018, and it was administered an additional time in March 2019 after the intervention in February 2019. RWS resident data were deidentified by the research coordinator, and all other investigators were blinded to the participants' data. Results were analyzed using a single factor ANOVA.

###### Results:

Overall, scores slightly improved from July to December 2018. Residents preferred time alone for personal life activity vs group activity engagement when given the choice. Resident buy-in was improved with focus on wellness, not burnout, and action instead of talking. A resident wellness officer was selected as part of the resident council.

###### Conclusion:

Challenges to this project included the lack of support from fellow residents and faculty for residents having allowed time off and difficulties scheduling GME-wide AIAMC planning meetings. Next steps include expansion of the program to address faculty wellness, as well as further development and support of program-specific wellness initiatives.

**Table:** PROJECT MANAGEMENT PLAN – “We” for Wellness

Vision Statement	Our vision is to create an environment that fosters the well-being of both our learners and our instructors. We strive to create a safe, inclusive, and supportive training environment for all involved.
Team Objectives	An objective for this project was to decrease resident burnout, as measured by the Wayne State University Resident Wellness Scale, by 5%, from our baseline of 3.59 to 3.77 over a period of 9 months by giving residents a half-day of dedicated wellness time. Another objective was to encourage program-specific wellness initiatives within each of our residency programs.
Success Factors	The most successful parts of our work were residents' recognition of efforts made for resident wellness and the appreciation of the wellness half-day. We were inspired by buy-in from residents when we listened to their desire for autonomy.
Barriers	The largest barriers encountered were time constraints around meeting, scheduling, and planning our events and the amount of time dedicated to our group. We worked to overcome these barriers by engaging appropriate administrative staff, learning who the key schedulers were at each program, and scheduling meetings well in advance.
Lessons Learned	The single most important piece of advice to provide another team embarking on a similar initiative is don't underestimate the value of having your whole planning committee together on a regular basis; preplan these meetings well in advance.

## Guthrie – Robert Packer Hospital, Sayre, PA

### Improving Well-Being and Work-Life Balance of Residents

#### Salman Khan, MD; Mahin Rehman, DO; Sheela Prabhu, MD; Victor Kolade, MD; John Pamula, MD; Tamara Davenport; Laura Fitzgerald

##### 

###### Background:

Physician burnout is a national phenomenon that leads to poor quality of care, errors, and diminished professionalism. Healthcare professionals are at risk, as burnout may raise their risk of suicide, and they may provide suboptimal care that results in lower patient satisfaction. Our goal for this project was to improve the well-being of our internal medicine residents by identifying stressors, reducing stress, and providing interventions.

###### Methods:

A baseline Maslach Burnout Inventory (MBI) was administered to internal medicine residents. Interventions were implemented in fall 2017, spring 2018, and spring 2019. Interventions included faculty-led interventions, GME activities, wellness with psychology sessions, an EAP, a program director dinner, a group chat on WhatsApp, a pizza night, recreational sport activities, and a gala. Periodic MBI surveys were administered following interventions. We also developed a Wellness Interventions Feedback Questionnaire to evaluate if interventions were enjoyable and a Resident-Led Intervention Questionnaire to see what residents wanted, given the choice.

###### Results:

Residents improved and stayed at improved levels. PGY2 participants were identified as having the highest levels of burnout. The WhatsApp group chat intervention had the most favorable and sustained response.

###### Conclusion:

Limitations to this project included limited cohesiveness among residents, resulting in low turnout at events and the fact that the project was limited to a small committee. Next steps include expanding the project to include internal medicine faculty and other residencies, developing a wellness curriculum for faculty and incoming classes, and implementing quarterly wellness activities.

**Table:** PROJECT MANAGEMENT PLAN – Improving Well-Being and Work-Life Balance of Residents

Vision Statement	Our vison is to foster a learning environment that promotes physician well-being, improve resiliency in our new physicians and find effective methods of reducing stress and burnout, and enhance our culture of wellness and better our patient care through bettering ourselves.
Team Objectives	Objectives for this project were to use the concept model of human coping reservoir identify stressors in residents via survey implement interventions involving the promotion of well-being and perform periodic assessments
Success Factors	The most successful part of our work was its completion. We were inspired by everyone's accomplishments presented at the other meetings.
Barriers	The largest barriers encountered were time management and resident turnout at the interventions. We worked to overcome these barriers by dedicating a rotation for the PGY2 participants to carry out the remainder of this project. We also recruited a PGY1 to facilitate the events and aid in data collection and the social promotion of well-being.
Lessons Learned	The single most important piece of advice to provide another team embarking on a similar initiative is to form a committee to help implement a plan and have events frequently.

## Hackensack Meridian Health/Jersey Shore University Medical Center, Neptune, NJ

### Resiliency in Residency – A “SHORE” Thing

#### Melissa Calt, MD; Nicole Fiore, MD; Amy Frieman, MD, MBA; Srividya Naganathan, MD; David Kountz, MD, MBA; Yen-Hong Kuo, PhD; Paul Schwartzberg, DO, MBA

##### 

###### Background:

Increasing rates of resident depression, suicide, and burnout highlight the importance of a curriculum directed toward resident well-being. This project aimed to determine the effectiveness of a new wellness program at our institution, The S.H.O.RE (Stress & Health Optimization for REsidents) Program, run by peer resident wellness champions from each department. We hypothesized that providing 3 months of weekly protected time for residents to learn and practice wellness exercises would help enhance their well-being and resilience, as well as their wellness, degree of burnout, and professional fulfillment.

###### Methods:

Residents in various programs at Hackensack Meridian Health, including OB/GYN, pediatrics, internal medicine, podiatry, surgery, pharmacy (Jersey Shore University Medical Center, Neptune, NJ), family medicine, and psychiatry (Ocean Medical Center, Brick, NJ), participated in brief wellness sessions on a weekly basis for approximately 12 weeks during protected didactic time. Each session included 3 wellness exercises and was run by a peer resident wellness champion from the department. The curriculum was focused on evidence-based, short, and effective exercises that decrease burnout and increase resiliency and happiness. The Stanford Physician Wellness Survey, a validated tool focusing on physician wellness, burnout, and professional fulfillment, was administered at the beginning and end of the program. Seventy-five presurveys and 41 postsurveys were completed, and 29 pairs were analyzed for changes. The outcome measurements included professional fulfillment, emotional exhaustion, interpersonal disengagement, and self-defined burnout.

###### Results:

Approximately 27% of residents reported definitely burning out, and the majority felt emotionally exhausted. There was no difference in distribution (presurveys and postsurveys) for emotional exhaustion (*P*=0.876) and self-defined burnout (*P*=0.767). After reviewing results from 29 residents who completed both the presurvey and postsurvey, 14 (48.3%) did not change their status of burnout, and 5 (17.2%) reported some improvement (I don't feel burned out).

###### Conclusion:

This program brought more awareness to the issue of burnout and resiliency in residency. To sustain this program, we plan to continue using our resident wellness champions as a cost-effective method to teach wellness and resilience skills. We also hope to obtain additional funding from our Medical Executive Committee and president of our medical and dental staff to expand the project on a larger scale and include physicians and staff. Our next steps include comparing postsurvey responses based on number of sessions attended, year of training, and subspecialty; developing new curriculum focusing on areas residents liked; continuing to use resident champions to train other residents; seeking outside consultants and resources; and developing more group wellness activities.

**Table:** PROJECT MANAGEMENT PLAN – Resiliency in Residency – A “SHORE” Thing

Vision Statement	Our vision is that resilience and wellness will be incorporated and integrated into all aspects of our training environment. Our mission is to foster a culture of well-being for our resident physicians and fellows by creating a curriculum based on the needs of our trainees to enhance and maintain their resilience and wellness. The curriculum will provide an array of services designed to encourage healthy lifestyles, promote optimal work-life balance, and build resilience. The knowledge, skills, and attitudes obtained by our house staff through this program will prevent compassion fatigue and serve as an essential foundation for lifelong physical and mental well-being for themselves and their patients.
Team Objectives	Our objective was to investigate whether 3 months of weekly protected time for residents to learn and practice wellness activities improved their wellness, degree of burnout, and professional fulfillment.
Success Factors	The most successful part of our work was gathering all our residents together in one room for a kickoff event during which they were asked to complete a survey either online or in paper form. In addition, we brought more awareness to the issue of burnout and resiliency in residency and emphasized the importance of wellness through grand rounds, the kickoff event, and the 12-week program. Overall, this project was successful in introducing wellness tools to our residents. We were inspired by the active interest of our residents across subspecialties in this wellness initiative and other studies that have shown positive outcomes from implementing a wellness program.
Barriers	Barriers included the following: Compliance of residents with the program Program ending during the winter (associated with higher rates of depression) Postsurveys completed during the middle of the year when residents may be more burned out Potential variations in the implementation style of the wellness activities between subspecialties due to each subspecialty having a different wellness champion Inability to compare all the data due to less than anticipated participation on postsurveys We tried to overcome some of these barriers by creating a standardized training manual for all the resident champions to follow. We also sent email reminders to our residents to complete the postsurveys.
Lessons Learned	The single most important piece of advice to provide another team embarking on a similar initiative is to implement a closing session. The closing session would be an opportunity, similar to the kickoff, to gather residents in a group setting to complete the postsurvey.

## HCA South Atlantic Division, Charleston, SC

### Faculty Understanding Resident Burnout

#### MG Flynn; J Johnson; L McMann; S Livingston; L Hart

##### 

###### Background:

Burnout rates are particularly high in trainees and are reported to be higher in female physicians, yet active strategies to address this problem are relatively new. Concerns about mistakes related to resident duty hours have received national media attention, but resident and wellness burnout issues are more complex than simply altering duty hours. In fact, duty hour restrictions require residents to complete their work in a shortened time frame, which could add to residents' stress. Rates of burnout among faculty and program directors are lower than in residents, but we questioned the ability of distressed faculty to identify signs of burnout or to address these issues in their residents. Research shows that 27% of internal medicine program directors reported emotional exhaustion, with 28.7% reporting burnout. In addition, burnout scores (emotional exhaustion, depersonalization, and lack of personal accomplishment) were highest in residents/fellows and medical students, but the differences between trainees and early career physicians were negligible. The ACGME has focused a portion of the required curriculum on addressing resident wellness/burnout concerns. However, research that addressees the approach of using faculty to identify and address resident burnout is insufficient, and efforts to further raise awareness and mitigate burnout among residents and early career physicians may be needed. The aims of our project were to (1) assess baseline knowledge of burnout among faculty, (2) quantify existing burnout among faculty and residents, (3) provide faculty training to recognize signs of burnout and implement strategies for burnout reduction, and (4) compare baseline and posttraining burnout among faculty and residents.

###### Methods:

Faculty and residents at 4 GME sites in HCA South Atlantic Division were invited to complete the expanded 9-item Well-Being Index (WBI) online. GME faculty members were also invited to complete an online knowledge check to assess their knowledge about burnout and to attend well-being workshops delivered by behavioral health specialists. The 75- to 90-minute workshop detailed and addressed signs of burnout, methods and strategies for managing burnout, and actions to take for residents who experience burnout. Faculty attending the workshops were invited to complete the postworkshop knowledge check within approximately 1 week. Both faculty and residents will complete the WBI again 3 months after the workshops.

###### Results:

Sixty-eight faculty members and 83 residents completed the WBI. A higher proportion of faculty were at risk than residents (42.6% vs 27.2%), according to respective WBI thresholds (physicians WBI ≥3; residents WBI ≥5). A higher proportion of PGY1 residents (39.9%) were at risk compared with other resident cohorts, and female residents and faculty were more likely to have at-risk threshold scores than male residents and faculty. Eighty faculty members completed the knowledge check quiz preworkshop, with an average score of 70% correct, and 36 faculty members completed the quiz postworkshop, with an average score of 84% correct.

###### Conclusion:

Research has shown that faculty and residents with WBI scores above their respective thresholds have greater risk for recently reported medical error, suicide ideation, poor mental quality of life, burnout, and severe fatigue. Our preliminary findings support our decision to train the faculty to recognize signs of burnout. We will invite faculty and residents to complete postworkshop surveys roughly 3 months after the presurveys have been completed.

## HealthPartners Institute, Minneapolis, MN

### Assessment of Resident Well-Being

#### Cullen Hegarty, MD; Mary Wagner, MD; Amy Borys, MD; Sarah Baker, MD; Kelly Frisch, MD; Cecily Spencer; Rebecca Rossom, MD, MS; Christopher Leonard, MD; Chelsey Sand, MD; Brooke Campbell; Jonathan Sellman, MD; Felix Ankel, MD; et al

##### 

###### Background:

Residency is a great time of learning and growth, but it is also a great time of stress. Studies have shown medical students and residents have a high percentage of depression, and a significant number of residents each year think about suicide—with suicide being the second leading cause of death for residents. Despite knowing this information and having programs promoting wellness, we wonder if more needs to be done to assess resident well-being during training to get at-risk residents the help they need before it is too late. Our primary aim was to incorporate a well-being self-assessment into each resident's semiannual evaluation process. Our secondary aims were to (1) trial having residents visit our hospital resiliency center and (2) create a section of the GME website dedicated to wellness and key resources for residents (eg, resident assistance program, wellness center).

###### Methods:

In 2018, the emergency medicine PGY1 and PGY2 residents completed the Wayne State Resident Wellness Scale self-assessment prior to their semiannual program evaluations and visited our hospital resiliency center. A wellness section on the GME website was created to highlight and house wellness resources for residents. We asked the residents to share their thoughts on these areas through an optional SurveyMonkey survey.

###### Results:

Fifty-five percent of the residents felt doing the well-being self-assessment was helpful, offering positive feedback such as “good to think about wellness/bring to awareness,” “It was a good way to reflect on my wellness and what coping skills I like to use,” and “I liked how it addressed different aspects of wellness and made me pay attention to things I maybe wouldn't think of as dimensions of wellness (diet, exercise, how I feel about my patients and how I feel about my work).” Seventy-two percent of the residents thought visiting the hospital resiliency center was helpful. Supportive comments included, “Great thing to do once so people know what kind of support is available there and can have return visits if they feel it is helpful,” “I like how calm it is. I like that there was a structured time set aside to address wellness,” and “good exposure to some resources and a nice area to de-stress.” Sixty-three percent of residents thought requiring residents to visit the resiliency center for a 30-minute orientation once during their intern year would be helpful to make them aware that it exists and to learn more about available resources.

###### Conclusion:

Emergency medicine has formally incorporated the Wayne State Resident Wellness Scale into the semiannual evaluation process, and GME will recommend that other programs do as well. Emergency medicine also now requires interns to visit the hospital resiliency center for a 30-minute orientation session, and other programs are considering this requirement as well. After the initiation of our project, the ACGME introduced new common program requirements effective July 2019, including a well-being requirement for programs to provide access to appropriate tools for self-screening.

**Table:** PROJECT MANAGEMENT PLAN – Assessment of Resident Well-Being

Vision Statement	At HealthPartners, we aim to assess our residents' well-being on a regular basis and support them in any way we can during their training time with us.
Team Objectives	We plan to incorporate a well-being survey into each resident's semiannual evaluation process and use the results to get them additional help or resources they may need. Our organization is aware of the data regarding depression and suicide in resident trainees, and we want to focus efforts on assessing and helping our residents.
Success Factors	The most successful parts of our work were implementing the well-being self-assessments implementing resident visits to our hospital resiliency center adding a wellness tab/section to the GME website getting feedback from residents on our project completing our project We were inspired by the willingness of the hospital to help us with this project.
Barriers	The largest barrier encountered was that, due to schedules/meeting times, emergency medicine was the only program that participated in the project from start to finish. In the end, however, we found that having only one residency program trial our project worked well. Emergency medicine was the right size program for us to try our methods and get feedback that we can now use throughout our GME system. Another limitation was that we did not get feedback regarding the wellness resource section of the GME website.
Lessons Learned	The single most important piece of advice to provide another team embarking on a similar initiative is to keep the project size and time frame realistic. We completed our project because we kept the focus and trial group small. Other advice is to get the key hospital personnel involved (in our case, the director of the health and wellness center) and to get GME buy-in for the project, as they were a great support for us.

## HonorHealth, Scottsdale, AZ

### Building Resiliency With Empathy Training

#### W Ellis, DHEd, MC, LPC; C Kegowicz, MD; P Radhakrishnan, MD; S Snell, MD; A DeGrio, MD; K Gosselin, PhD; A Brandon, BS; S Easterly, MS IV

##### 

###### Background:

Physician burnout and professional dissatisfaction have reached epidemic proportions in healthcare, and addressing these issues in proactive, sustainable ways is a priority of multiple medical organizations including the ACGME. Evidence supports the benefits of improved personal resiliency and enhanced social connectedness in the reduction of burnout risk. Our project assumption was that enhanced ability to empathize and build relationships contributes to the well-being of caregivers by increasing their overall resilience, flexibility, social connectedness, and ability to self-regulate. Our project goal was to develop, implement, and cultivate a culture of resiliency throughout HonorHealth and its residency programs.

###### Methods:

Our assessment and intervention plan initially targeted GME across all programs, but, due to availability conflicts, we made use of a convenience sample. Participating residents took pretests to establish baselines for each training year (PGY1, 2, and 3) utilizing three measures: the Connor-Davidson Resiliency Scale, the Toronto Empathy Questionnaire, and the Multidimensional Scale of Perceived Social Support. One month after an empathy educational training workshop, data from these scales were again collected via electronic survey. Despite our best efforts, we only had 10 paired presurvey and postsurvey respondent samples for data analysis (∼20% response rate). Of these, 8 paired response sets were from the 19 residents who participated in the educational workshop.

###### Results:

During our project, we learned that GME programs each had a unique set of needs and thus required efforts to address their specific local cultures and workflows. Additionally, attempts to target interventions that focused on individual prevention and behavioral changes were met with a great deal of pushback from the residents and were seen as “revictimizing the victim.” Residents consistently expressed a request to “fix the broken system not us.” This anticipatory disengagement fit with issues being addressed at the institutional level. Due to these discoveries and other data collection issues, we shifted our plan to include looking at our sample's baseline data which we could then incorporate into our larger institutional initiatives, collaborations, and efforts. The small sample size also affected our ability to detect differences on the scales before and after the workshop. However, the increase in empathy scores of the 8 people who participated in the workshop was statistically significant (*P*=0.007). At the training and days later, participants agreed that they enjoyed and benefitted from the training workshop.

###### Conclusion:

The project showed that it is possible to create educational interventions that foster and enhance physician empathy and also raised the question of how to foster individuals' intrinsic motivation for personal prevention of burnout and disengagement. What we initially set out to do yielded a number of insights and learnings that provoked a systemwide transformative initiative, including the formation of the GMEC Wellbeing Work Group. This work has not only informed but sparked the development of our institutionwide well-being initiatives, not only for GME programs but also for all caregivers. We now see tremendous energy and enthusiasm around well-being efforts across all departments and among system stakeholders. This work is in harmony with our system's strategic goals, values, and vision.

**Table:** PROJECT MANAGEMENT PLAN – Building Resiliency With Empathy Training

Vision Statement	We are committed to advancing and supporting a culture of well-being across our institution and specifically in the newest generation of medical providers. We firmly believe that to successfully champion a physician well-being culture, we must articulate, develop, and implement a proactive, sustainable approach to nourishing a culture that values and enhances strengths, balance, resilience, and well-defined resources as opposed to simply trying to fix accumulated stress and burnout after the fact.
Team Objectives	Our team objectives were as follows: Make HonorHealth the employer of choice Develop an enhanced program for prevention of caregiver burnout Develop processes and resources for caregiver crisis management Identify and map a resident-centric well-being program for all GME programs Create and implement a sustainable empathy education/training program Develop accessible prevention and treatment resources Enhance resident physician and caregiver engagement and alignment Prevent caregiver burnout, thereby reducing adverse patient outcomes and improving patient and provider satisfaction Promote a trauma-informed culture and response to secondary trauma contributing to caregiver burnout Promote and support healthy teamwork and a compassionate culture
Success Factors	The most successful part of our work was gaining a commitment from our institution for a systemwide sustainable, scalable well-being program. We were inspired by everyone's unique stories of resilience and collaboration and their desire to build upon and enhance these efforts.
Barriers	The largest barrier encountered was lack of accessibility to and time with residents due to their varying schedules. These time constraints led to our pilot project having a revised timeline and schedule and a smaller sample of participants than anticipated. We worked to overcome these barriers by soliciting buy-in and support from program directors and House Council leadership. Other limitations included survey fatigue, varying motivation for individual prevention improvement efforts, and a potential ceiling effect with the Connor-Davidson Resiliency Scale baseline mean score, which may have limited the ability to see significant change, in addition to a small sample size.
Lessons Learned	Important advice to provide another team embarking on a similar initiative is to use qualitative vs quantitative data collection methods whenever possible. Honor and value the multiple existing resources as well as the diversities encountered. be open and willing to adapt and change initial plans as work unfolds. openly communicate and demonstrate action based upon feedback from stakeholders. Keep everyone informed. continually reassess and enhance engagement. remember that a sense of community and connectedness is the underpinning of success and the key to project sustainability.

## Main Line Health System, Bryn Mawr, PA

### Improving Resident Wellness with FirstCall Assistance

#### Sandra Ross, LSW; Kelly Campanile, PsyD; Katherine Corvi, PsyD; Jonah Klein, MD; Sharon Iannucci, Mgr GME; April Lockley, DO; Vishal Shah, DO; Daniel Buckalew, Mgr Health and Productivity; Barry D. Mann, MD; Chinwe Onyekere, MPH; Joseph A. Greco, MD

##### 

###### Background:

High stress and burnout among resident trainees affect mental health, self-care, and patient care and can lead to increased rates of suicidal ideation, stress, and depression. The Main Line Health System (MLHS) 2018 Strategic Plan emphasized the need to (1) plan for the workforce of the future, (2) foster a culture of lifelong learning and sharing, and (3) maximize the potential of residency and fellowship programs. MLHS believes that wellness will propagate patient satisfaction, safety, and quality. Decreased shift length, stress management and self-care sessions, communication skills education, and structural interventions have been shown to decrease burnout, emotional exhaustion, and depersonalization in both attending and resident physicians. Several institutions have EAPs available to residents, but whether residents use or are aware of these programs, as well as if these programs are adaptable to residents' needs or adequately address burnout and wellness, is unknown. The purpose of our project was to connect residents and fellows with services and resources available through the MLHS EAP, FirstCall. Our aims were to (1) assess the degree of burnout among residents and fellows of varying specialties, (2) assess the awareness of services that are offered by FirstCall, and (3) determine if the FirstCall EAP can adequately address the needs of trainees to decrease burnout and enhance wellness.

###### Methods:

To educate residents and fellows about available EAP resources, we planned for FirstCall to create a flyer promoting services most relevant to trainees and to direct trainees to their clinical coordinator for high-touch follow-up interactions. We evaluated residents' awareness of services offered through FirstCall. To obtain baseline burnout/wellness data, we asked residents to complete the Areas of Worklife Survey and Maslach Burnout Inventory. Residency programs participating were family medicine, internal medicine, general surgery, OB/GYN, podiatry, and radiology.

###### Results:

Less than 50% of trainees were aware of FirstCall services, and less than 75% of trainees were aware of behavioral health support and conflict mediation. Trainees from all programs (50%-82%) agreed or strongly agreed that they have so much work to do that it takes them away from personal interests. Sixty-five percent of trainees reported feeling emotionally drained from their work at least a few times a month, and 39% reported feeling this way at least a few times a week. Seventeen percent of internal medicine residents said they're callous every day. The results of the wellness survey also revealed some of residents' alarming coping mechanisms, such as intentionally becoming more callous.

###### Conclusion:

Treating patients like impersonal objects and increasingly exhibiting callousness are common behaviors among trainees, yet trainees are mostly unaware of available resources. Despite depersonalization and burnout, trainees feel exhilarated and a sense of accomplishment when working directly with patients. Going forward, FirstCall will create trainee-specific presentations and offer them at orientation and various other times throughout the year. We plan to continually assess the impact FirstCall has on trainee burnout and wellness and to enhance GME expertise within the FirstCall staff. Teaching residents in a system that promotes burnout and not wellness encourages residents to establish practice elsewhere upon graduation. However, when trained in a demanding yet nurturing and responsive continuous learning environment, residents are more likely to perpetuate self-care practices and stay locally on the medical staff.

**Table:** PROJECT MANAGEMENT PLAN – Improving Resident Wellness with FirstCall Assistance

Vision Statement	Main Line Health System (MLHS) is committed to a continuous learning environment that must be nurturing and attentive to promote wellness. MLHS will be a thought leader in this area and will take measures to decrease burnout and promote wellness in residents, faculty, and health system employees. MLHS believes that wellness will propagate safety and quality.
Team Objectives	The purpose of our project was to assess, adapt, and ultimately connect our residents and fellows with the MLHS EAP, FirstCall.
Success Factors	The most successful parts of our work were administering the burnout surveys to residents and beginning a healthy dialogue with human resources (HR) about resident-specific needs within the EAP. We were inspired by the willingness of residents to be honest about their levels of burnout in their responses to the survey.
Barriers	The largest barrier we encountered was our inability to demonstrate the impact of our interventions within the time frame provided. We encountered barriers with FirstCall implementing some of our suggested interventions, including tracking resident use of services and issuing flyers and presentations specific to residents. FirstCall was unable to meet with and educate all residency and fellowship programs about their services during the project timeline. We are working to overcome these barriers by organizing feedback sessions between GME and HR/EAP. The internal medicine program hired a wellness director who should help bridge the perceived gap between these two departments.
Lessons Learned	The single most important piece of advice to provide another team embarking on a similar initiative is to encourage residents to assume leadership roles in the project and to ensure that program directors designate time for residents to participate in meetings.

## Maine Medical Center, Portland, ME

### Investigating Gender Bias at an Independent Academic Medical Center

#### Kalli Varaklis, MD, MSEd; Katherine Rizzolo, MD; Thomas van der Kloot, MD; Bob Bing-You, MD, MBA, MSEd

##### 

###### Background:

In 2017, Maine Medical Center (MMC) underwent a CLER site visit by the ACGME. An unexpected finding in the report was, “In the group setting, several residents from a variety of programs, confirmed by faculty, reported that there is significant gender bias at MMC, such that female residents are treated worse than their male counterparts.” Multiple published reports exist that describe the effect of gender bias in graduate medical education. MMC is an independent academic medical center in Portland, ME, with approximately 276 residents and fellows in 24 training programs. The objectives of the project were to (1) gauge baseline resident perception of gender bias and its effect on wellness metrics and (2) develop a series of interventions to address perceived gender bias discrimination among house staff.

###### Methods:

Two new positions were created, a Title IX officer and a GME social worker. Senior nursing leadership was engaged for a nursing response, and an e-learn training on sexual harassment was implemented for more than 19,000 employees. Engagement with the new MMC Diversity/Equity/Inclusion (D/E/I) Committee commenced, including a D/E/I session at intern orientation. The DIO met with resident groups from 20 of 22 training programs and asked one scripted question regarding potential gender bias discrimination. A validated survey exploring gender bias in surgical residents was modified and sent to residents to assess the incidence of experienced and observed perceived gender bias, as well as the effect on wellness metrics (burnout, stress, ability to work in an interprofessional team, clinical work quality, and job satisfaction). The DIO met with residents and fellows again postintervention, and a resident forum was held.

###### Results:

One hundred and nineteen trainees (45.4%) completed the baseline survey, and 55 trainees (19.9%) completed the postintervention survey. The perception of gender bias was not different before and after the intervention. However, we saw a trend toward an improved perception of quality of clinical work being negatively affected by perceived gender bias after interventions.

###### Conclusion:

We did not anticipate the response rate for the initial survey, the degree of gender bias perception reported by female house staff, or the extent to which perceived gender bias negatively impacted wellness metrics. We plan to transition the project into the newly created institutional D/E/I Committee for oversight and applicability of lessons learned to the non-GME community. We also plan to expand exposure of our project at orientation, including microaggression training, and continue to roll out the newly created Title IX officer. Finally, we intend to publish our findings and modified validated survey for use in other institutions and administer a follow-up survey in 2 years.

**Table:** PROJECT MANAGEMENT PLAN – Investigating Gender Bias at an Independent Academic Medical Center

Vision Statement	Our vision is that our clinical learning environment will be free of gender bias such that every member of the healthcare team is treated with respect and equity.
Team Objectives	Our mission is to provide a better institutional understanding of the scope and nature of gender inequity experienced by physician trainees at Maine Medical Center (MMC). This knowledge will allow us to design targeted interventions that can be shared with other interprofessional and interdisciplinary leaders in our institution to investigate the experience and extent of gender bias among other MMC stakeholder groups.
Success Factors	The most successful aspects of the project were engagement from trainees overall positive response from institutional leaders trend toward improved perception of quality of clinical work being negatively affected by perceived gender bias after interventions creation of 2 new positions: Title IX officer and dedicated GME social worker
Barriers	Barriers and limitations included rare response from leaders, either undermining effort or discounting report lack of local expertise significantly decreased response rate from trainees in the postintervention survey bias from not sampling the same trainee group one year to the next
Lessons Learned	The project taught us to communicate early and frequently with senior leaders understand that GME learners are employees and that human resources needs to be involved early and often in a sensitive topic such as this one engage local experts

## Monmouth Medical Center – RWJBarnabas Health, Long Branch, NJ

### Monmouth Medical Center Well-Being Program

#### Dr. Joseph Jaeger; Pranoy Mohapatra; Julie Villa, RN; Juliet M Gossett, MBA; Rose Polasky, RN; Dr. Jim Romer; Dr. Margaret Eng; Dr. Margaret Fisher; Dr. Gregory Greco; Dr. Robert Graebe; Dr. Richard Ruchman; Dr. Raymond Simmons; Dr. Steve Paragioudakis, MBA; Yasmin Ahmed; Dr. Ilyas Chiali; Dr. Amrit Basnet; Dr. Sai Koyoda; Shreeja Kadakia; Dr. Ethan Paulin; Dr. Stephanie Scianni

##### 

###### Background:

Burnout, a mixture of exhaustion and depersonalization, is prevalent among clinical practitioners and leads to employee turnover, adverse events, and increased risk in patient care. Forty-five percent of residents experience at least one major symptom of burnout, with wide variability by specialty, anxiety, and organizational/familial support structure, and many practitioners even come to regret their career choice. We aimed to implement a hospitalwide well-being initiative that adequately met the needs of all clinical practitioners. Our objectives were to (1) increase the collective well-being score by 10% of at least 70% of measured groups and (2) incorporate and destigmatize wellness through monthly correspondence and education.

###### Methods:

Our plans were to administer the Mayo Well-Being Index (WBI) 1 month after orientation, and quarterly thereafter, to all MMC residents, nurses, and staff physicians, noting residents' specialties, years, and hours per week; physicians' specialties, ages, percentages of time spent on administrative tasks, and whether they spend most of their time on inpatient or outpatient duties; and nurses' employment status (part time or full time), hours per week, and highest levels of education. We implemented presentations on BHealthy, the in-house wellness program, to all groups and created a resident lounge that is stocked with light foods during hours the cafeteria is closed. We also replaced equipment in the fitness facility to align with employees' needs and are working to increase access to the facility by expanding hours and improving surveillance and security. Furthermore, we improved access to the EAP and financial services and now offer presentations and services specifically for residents.

###### Results:

We found that access to healthy and readily available food options is a high priority for residents and discovered a lack of awareness of existing initiatives, options, and rewards offered through BHealthy. We sampled 120 residents, 856 physicians, and 937 nurses. Data from the WBI will be benchmarked against national averages and reassessed quarterly.

###### Conclusion:

Fostering employee health and well-being is an ongoing process, and each employee must feel that their individual needs are being addressed in an earnest and honest manner. Moving forward, we plan to put a well-defined communication tree in place to disburse information and to continue to administer the WBI with support from leaders and representative champions.

**Table:** PROJECT MANAGEMENT PLAN – Monmouth Medical Center Well-Being Program

Vision Statement	Our vision is to foster a medical education experience committed to an encouraging, supportive, and healthy pursuit of knowledge, learning, training, and growth. As a healthcare organization, we are devoted to a healthier community that acknowledges and is prepared to address well-being, including among our own residents and staff.
Team Objectives	Our objectives were to increase the collective well-being score by 10% of at least 70% of measured groups incorporate and destigmatize wellness through monthly correspondence and education build a scalable program extending throughout the hospital, and eventually the system, with support from physician leaders and the C-suite
Success Factors	The most successful parts of our work were the increased participation, awareness, and specificity of wellness efforts, as well as the investment in infrastructure to support a sustainable wellness culture. We were inspired by making progress on our milestones.
Barriers	The largest barrier we encountered was keeping a consistent group moving forward on multiple projects at once. Accountability was difficult to maintain, and projects would slow down and hit barriers at different points across the initiative, many times out of the control of the steering committee's hands. We worked to overcome these difficulties by using several smaller, more focused meetings rather than steering committee meetings which often involved different constituents each time, resulting in repetition of past meeting information and competing priorities/agendas.
Lessons Learned	The single most important piece of advice to provide another team embarking on a similar initiative is that broad, hospitalwide initiatives are great for the culture, but information still must be delivered specifically and curated toward intended targets. Ensure leadership is committed and lends significance, finance, and importance to the efforts.

## Ochsner Health System, New Orleans, LA

### Innovating Resident Well-Being

#### Stuart Hart, MD; Ronald Amedee, MD; Shelly Monks; William “Tex” Walker

##### 

###### Background:

This resident well-being project aimed to assess well-being, resilience, and resource awareness of residents. We aimed to increase the number of residents aware of supportive resources by 50% and to improve scores on Maslach Burnout Inventory (MBI) measures.

###### Methods:

In spring 2017, we discovered that 40% of residents were unaware of existing supportive resources. During summer and fall 2017, Ochsner Health System initiated a wellness task force for physicians and midlevel providers, deployed the Maslach Burnout Inventory (MBI), and assembled focused listening groups, but residents/fellows were excluded. Ochsner GME then established a team to assess and improve resident well-being across the system, deployed the MBI to residents and fellows, and conducted resident forums to assess and prioritize potential strategies. The objective of our project was to improve the condition of resident well-being at Ochsner through the enhancement of wellness infrastructure, accessibility, and awareness. All residents were requested to participate in the project. Residents were sent a SurveyMonkey survey to gauge their awareness of wellness resources before and after the campaign and an anonymous survey link to complete the MBI.

###### Results:

MBI scores suggested some residents may be at risk, leading us to conclude that existing well-being screening was not effective in identifying at-risk residents. Resident scores on the MBI correlated directly to the tenure of resident experience. Resident MBI scores were worse than the national peer norms for emotional exhaustion and depersonalization early in tenure but better than national norms for personal accomplishment for all program levels. We implemented several interventions: revising resident onboarding to include information on available resources and where to reference them on the intranet; adding a priority link on the resident's Resident Management System homepage with wellness information; conducting DIO/assistant vice president rounding in resident areas; conducting resident well-being forums and assessing proposed strategies; improving the mechanism for dissemination of existing wellness resources to residents; and establishing an institutional Provider Wellness Office. Resident awareness of supportive resources increased from 60% preintervention to 74.6% postintervention.

###### Conclusion:

Interventions to promote wellness resources improved resident awareness of those resources, but the survey response rate was low. The awareness survey response rate was only 23%. Certain demographic characteristics of residents, such as lack of a local support system, correlated with adverse scores on the MBI, but the response rate was only 48%. Overall, resident participation in the initiatives, including focus groups, was low. Moving forward, we intend to deploy a number of additional interventions for the 2019-2020 class and repeat the MBI campaign to determine the effectiveness of the interventions.

**Table:** PROJECT MANAGEMENT PLAN – Innovating Resident Well-Being

Vision Statement	Our vision is to improve the condition of resident well-being at Ochsner through the enhancement of wellness infrastructure, accessibility, and awareness and to measure success through improvements in Maslach Burnout Inventory (MBI) measures.
Team Objectives	Our objectives were to administer an MBI baseline and postintervention survey implement wellness and awareness strategies administer a follow-up survey on resident awareness of wellness resources inventory all existing wellness strategies in programs
Success Factors	The most successful parts of our work were moving up the timeline for resident integration into the institution's provider wellness initiative and installing the new Office of Provider Wellness. We were inspired by resident sincerity and openness during focus groups.
Barriers	The largest barrier we encountered was a lack of resident interest/time in participating in the project. We worked to overcome this challenge by continuing to recruit participants and modifying focus group times to encourage participation. Another limitation was the low MBI and awareness survey response rates of 48% and 23%, respectively.
Lessons Learned	The single most important piece of advice to provide another team embarking on a similar initiative is to ensure protected time from other operational commitments to sustain project momentum.

## OhioHealth – Riverside Methodist Hospital, Columbus, OH

### Even Better Yet…Refining Resident-Led Initiatives to Successfully Mitigate Burnout

#### Emily Gorman, DO; Charlotte Venious, DO; Stephen Auciello, MD; Laurie Hommema, MD; Emily Stansbury; Anand Gupta, MBBS, MPH

##### 

###### Background:

In response to high levels of resident burnout in 2014 and 2015, the family medicine (FM) program at Riverside Methodist Hospital implemented a wellness curriculum targeting the 3 components of burnout. The curriculum focused on faculty-led improvement of the clinical learning environment and reduced dedicated didactic time to allow for resident-led wellness initiatives and activities. The curriculum successfully reduced FM resident burnout during the last 4 years, while burnout across medical education remained the same. This year, due to increased resident engagement, a wellness committee focused on improving the wellness curriculum was formed. Our objective was to continue to decrease burnout among the 18 FM residents by refining existing wellness initiatives as measured by the modified Maslach Burnout Inventory (mMBI).

###### Methods:

From 2012 to 2019, a resident well-being survey was distributed annually to all residents at our institution. The survey included an mMBI as well as questions addressing communication and working environment. Results from 2015 to 2019 were analyzed to track changes in burnout scores. This year's interventions included continued protected wellness time of 1 hour per block in lieu of didactics; 4 protected afternoons for scheduled events each year, as well as Doctor Days; more advanced planning of wellness events driven by the wellness committee; integration of office staff and faculty into events; and a refined Doctor Day policy that has less of a set schedule and is more flexible and individualized. We intentionally selected our events based on the 10 domains of well-being outlined by Stanford's Catherine Heaney, PhD—social connectedness, lifestyle behaviors, physical health, stress and resilience, emotional and mental health, purpose/meaning, sense of self, finance, spirituality/religiosity, and creativity—and we added an 11th domain of giving. Events included a giving tree for residents to contribute to patients during the holiday season; officewide volunteer and team-building events; ice skating with faculty, students, and residents; and self-reflection exercises for residents.

###### Results:

Refinement of the FM wellness curriculum resulted in continued reduction of FM resident burnout. The mean score on the mMBI decreased from 31 in 2018 to 27 in 2019. A score of ≥27 indicates at least a moderate level of burnout. The success of the FM model wellness curriculum was lateralized to other programs in medical education at Riverside.

###### Conclusion:

Our project was limited due to the survey being completed only once a year and anonymously. Despite the reduction in mean score on the mMBI, many residents still experience burnout. Going forward, we will establish a schedule of successful events for future academic years, utilize the wellness committee for continuous improvement, and move to a more real-time measurement tool.

## OhioHealth – Riverside Methodist Hospital, Columbus, OH

### Internal Medicine and Preliminary Medicine/Transitional Year Wellness Interventions

#### Deep Patel, DO; David Arnold, DO; Stephen Auciello, MD; Sara Sukalich, MD; Emily Stansbury; Anand Gupta, MBBS, MPH; NI VI IM/PM/TY Team

##### 

###### Background:

Wellness in medical education has been a focus at Riverside Methodist Hospital (RMH) for the last several years as the modified Maslach Burnout Inventory (mMBI) revealed moderate levels of burnout in all residency programs. During the last year, the internal medicine (IM), preliminary medicine (PM), and transitional year (TY) residency programs have embraced a comprehensive focus on wellness and have implemented multiple resident-led interventions. The aim of our project was to decrease burnout among IM and PM/TY residents as demonstrated by improved mMBI scores by implementing resident-driven interventions.

###### Methods:

RMH has been tracking resident burnout with an anonymous and voluntary annual survey that features an mMBI and communication and peer support questions. During the last 2 years, IM implemented several curricular changes and wellness interventions to provide a more comprehensive focus on resident well-being. The PM and TY programs usually have the highest burnout scores of any residency program at RMH, and previous efforts have focused on including them in IM and medical education wellness interventions. For academic year 2018-2019, we implemented a monthly PM/TY reflective group. Interventions involving IM and PM/TY included a point-based resident incentive system to reward wellness and scholarly activity, institution of a fatigue mitigation/transition of care policy with travel reimbursement for residents too fatigued to drive home after 24-hour or night shifts, implementation of resiliency rounds after ICU rotations to help residents decompress and process thoughts and struggles, monthly board game nights and social activities, and shifting scheduled didactic time to wellness efforts. IM targeted interventions included a 4+2 curriculum to make calls more predictable and separate inpatient/outpatient responsibilities, as well as quarterly wellness days to attend doctor appointments or handle other personal needs. The PM/TY targeted intervention was a monthly reflective lunch group focusing on group identity and the unique challenges of PM and TY residents.

###### Results:

Comprehensive support of wellness in a residency program with multiple targeted wellness interventions led to decreased mean mMBI scores. Involvement of IM chief residents in wellness was a key component of successful interventions. Giving PM/TY residents a group identity may decrease burnout, but a lack of inclusion in interventions of other programs may increase burnout. Residents offered the following comments about the wellness interventions: “Med Ed should continue to do what it does now to make the workplace a little more like home, to feed us from time to time and host events for us to do outside the hospital;” “I feel that our half-day wellness days are very helpful in reducing burnout and giving the residents the feeling of a light at the end of the tunnel;” and “I think the most important aspect of maintaining a culture of wellness is ensuring that senior residents and attendings recognize when interns or those below them are overwhelmed and then attempt to help. Even if the senior can only help a little it is encouraging to have the support. I think that the residency program fosters this culture 99% of the time.” One resident also suggested we offer “continued focus on wellness and lasting interventions adjusted to each year's needs,” and another suggested that we “make sure we treat each other well, feed us from time to time, and have our backs if we need to battle the system that delays and frustrates our care for patients.”

###### Conclusion:

Many residents are now identifying systemic drivers of burnout that are much more challenging to change. We also experienced financial limitations and variability of resident and faculty buy-in. Moving forward, we plan to identify the interventions that made the most impact on wellness to ensure sustainability and to better engage faculty in wellness offerings.

## OhioHealth – Riverside Methodist Hospital, Columbus, OH

### Targeted Interventions to Improve Resident Well-Being

#### Marja Brolinson, MD; Karen D'Angelo, MD; Laurie Hommema, MD; Stephen Auciello, MD; Emily Stansbury; Anand Gupta, MBBS, MPH

##### 

###### Background:

During the medical education wellness efforts at Riverside Methodist Hospital (RMH), the OB/GYN residency program was noted to have the highest modified Maslach Burnout Inventory (mMBI) score among all programs, and residents reported that current interventions were not meeting their needs. These findings prompted a focused intervention in the OB/GYN residency, supported by medical education and led by faculty and residents in the OB/GYN program. Project objectives were to reduce OB/GYN resident burnout, enhance well-being, and evaluate the effectiveness of targeted program-specific and resident-driven interventions.

###### Methods:

RMH has been tracking resident burnout with an anonymous and voluntary annual survey that features the mMBI and communication and peer support questions. A pilot substudy consisting of multiple targeted interventions based on an initial focus group of residents was approved in September 2018 to address high levels of resident burnout among the 19 OB/GYN residents. A preintervention survey consisting of the Mayo Well-Being Index (WBI) and assessment of targeted interventions was conducted in September 2018. A postintervention survey was completed in January 2018 in conjunction with the annual mMBI survey. Interventions involved pager-free didactics during which covering attendings carried call pagers to allow residents protected time for educational events. A third resident was added to the previous 2-resident call team to help manage increasing volume, and a formal mentorship program was implemented in which residents were paired with attending mentors.

###### Results:

Ninety percent of respondents felt that their learning had been enhanced by pager-free didactics, and 92% of respondents felt that the quality of patient care had improved after adding an additional resident on call. The number of residents who stayed >1 hour after the end of their shift decreased from 100% to 46.1% (*P*=0.005). A statistically significant decrease in mean mMBI was seen in the OB/GYN residency program, and most residents reported that the targeted interventions enhanced learning, improved patient care, and assisted with future career preparations. Although the change in mean overall Mayo WBI was not significant, the percentage of residents who felt overwhelmed decreased, even though the postsurvey was administered during a stressful part of the academic year (53% vs 31% of respondents).

###### Conclusion:

We encountered limitations in that the initial Mayo WBI was administered after the mentorship and pager-free didactics interventions had already started. Additionally, the focus group revealed some hard truths and may have resulted in some residents being less truthful on surveys. Overall, however, the interventions created and sustained by program faculty and residents with medical education/organization support appear to be effective strategies to decrease burnout. We will sustain and refine these interventions based on resident feedback.

## OhioHealth – Riverside Methodist Hospital, Columbus, OH

### Better Me, Better *WE*

#### Stephen Auciello, MD; Sara Sukalich, MD, MEd; Laurie Hommema, MD; Charlotte Venious, DO; Deep Patel, DO; Emily Stansbury; Anand Gupta, MBBS, MPH

##### 

###### Background:

Wellness in medical education has been a focus at Riverside Methodist Hospital (RMH) for the last several years, but burnout levels worsened from 2012 through 2016 despite initial interventions. Communication breakdowns and perception of peer/faculty support were significantly correlated with burnout. In 2016, a more focused and comprehensive wellness curriculum in the family medicine (FM) program brought significant reductions in burnout, while other programs worsened or stayed the same. With this National Initiative, we lateralized the principles of the FM wellness curriculum across medical education at Riverside. This framework was based on principles of faculty-led change agents, resident champions who led program-level interventions, and action on resident feedback to reduce drivers of burnout. Our objectives were to create a wellness framework across medical education, support targeted wellness interventions and resident/faculty champions in each residency program, reduce burnout among residents at RMH, and enhance our culture of wellness within RMH medical education.

###### Methods:

Interventions included the creation of a wellness framework with resident and faculty representatives across medical education and within each program, consistent use of interdisciplinary resident time to gather feedback and support a culture of well-being, action on resident suggestions for enhancing well-being (more access to snacks and computers, protected study space), and engagement of residency faculty and teaching physicians in wellness offerings. We held the annual medical education Halloween party and have a newly constructed Medical Education Conference Center that includes an additional computer lab. We also deployed a yearly modified Maslach Burnout Inventory (mMBI) survey.

###### Results:

Our project resulted in decreased mMBI burnout scores and percentage of at-risk residents (from 74% in 2018 to 50% in 2019), enhanced perception of a culture of wellness among residents and faculty in medical education, improved resident perception of peer/faculty support and burnout recognition, and sustainable resident-owned interventions. An increased percentage of residents agreed or strongly agreed when prompted “My superiors and peers support and assist me when I am stressed or burned out,” and disagreed or strongly disagreed when prompted “Communication breakdowns that lead to delays are common in my work setting.” When comparing year 2019 with the combined responses of 2012 to 2018, a statistically significant (*P*=0.002) increased percentage of residents agreed or strongly agreed when prompted “My superiors and peers recognize stress and burnout in others.”

###### Conclusion:

A comprehensive wellness framework with faculty support, protected time for wellness, and resident-led interventions can lead to reductions in burnout rates and measurable cultural change. Although initial interventions may not be successful, long-term support of resident wellness can engage and excite residents to be an active part of this change. Faculty availability and interest varied by program, and we experienced other limitations such as financial constraints and addressing the work compression and role of residents in a large hospital system. Going forward, we will ensure successful interventions are sustainable, better engage faculty in wellness offerings and evaluation, and help enhance system-level support of wellness programs.

**Table:** PROJECT MANAGEMENT PLAN – Better Me, Better *WE*

Vision Statement	Our vision is to strengthen our culture of wellness in order to become the place where physicians want to teach, trainees want to learn, and people receive high-quality community-centered care. Our mission statement is to deliver a comprehensive and innovative wellness curriculum across medical education targeted to the needs of each residency program.
Team Objectives	The objectives of our project were to decrease burnout among residents and faculty offer a consistent and transparent wellness framework across medical education, driven by resident leadership and ideas and supported by faculty support targeted interventions within each residency program enhance awareness of burnout as a system/environmental issue and workplace hazard enhance our culture of wellness within medical education where impediments of wellness are recognized and faculty/residents work to remove the impediments
Success Factors	The most successful part of our work was engaging residents in a culture of residents. We saw a change in resident attitudes with this initiative compared to previous years of trying to reduce burnout. Residents across various programs became engaged in creating new wellness initiatives, diving deeper into existing issues and working to improve initiatives started by other programs. We were inspired by the success of some early interventions and watched as interest snowballed into action.
Barriers	The largest barrier encountered was the differences between the residency programs. Each program encountered unique challenges, and our wellness initiatives needed to be program-specific. We had inconsistent faculty support in some programs, and residents started to identify more systemic and hospital drivers of burnout outside of medical education, which will require more time and effort to fix. We worked to overcome these limitations by individualizing efforts and supporting our resident and faculty champions in each program.
Lessons Learned	The single most important piece of advice to provide another team embarking on a similar initiative is that meaningful change takes time and persistence. We were met with initial negativity and residents who were “burned out talking about burnout.” However, as we continued to ask our residents how to help them and acted on their suggestions, a spirit of friendly competition and a desire to engage in making things better emerged. We now have resident and faculty champions leading various interventions targeted to their needs, and a consistent support from medical education will keep that driving forward.

## Orlando Health, Orlando, FL

### Wellness at Orlando Health

#### K Ayesu, MD; Y Olivera Arencibia, MD; H Le, MD; M Madruga, MD; M Senne, PhD; M Griffin, MHA

##### 

###### Background:

Burnout among learners is reported to be ∼60% and rising, and there is an urgent need for academic programs to embark on initiatives to combat burnout and promote well-being. Adverse effects of burnout on physicians are far reaching and negatively impact patient and provider safety. Burnout has also been associated with decreased professionalism, and ACGME promotion of well-being highlights the seriousness of this problem. Prior to initiating our project, we had no database on the prevalence of burnout among learners and no direct GME engagement on this issue. The focus of our project was to (1) establish a baseline prevalence of burnout among learners and (2) promote programs that would enhance well-being. Our objective was to decrease physician burnout by 10% within 6 months, and in the process, promote resilience and a joyful working environment. We aimed to promote wellness across all residency/fellowship programs at Orlando Health (OH), spearheaded through GME leadership.

###### Methods:

Our methods included a baseline determination of burnout rates using the Mini-Z Burnout survey from the American Medical Association via New Innovations for 14 GME programs. We sent repeat surveys every 6 months to reassess. The director of physician wellness held group meetings and gave wellness and burnout lectures to all the individual programs. Individual programs were allowed to pursue program-specific approaches to address wellness and burnout. The GMEC passed a requirement for all learners to visit the director of wellness twice a year before program director evaluations. Residents identified causes and suggested solutions to reduce burnout, social events were held, and protected time was provided for internal medicine (IM) residents. We measured burnout rates preintervention and postintervention in addition to the effectiveness of program-specific strategies in coping with burnout and wellness.

###### Results:

Curricula and joint events were established in collaboration across GME programs to promote wellness. OH launched a wellness program website for physicians run by two physician coaches, and our GME team was involved in the production. We implemented 2 hours of protected time per month for wellness and resilience for IM residents. For IM residents, preintervention questionnaire completion rates were 100% for all PGY levels, and burnout rates for PGY1, PGY2, and PGY3 were 7% (1/15), 8% (1/13), and 25% (3/12), respectively. Postintervention questionnaire completion rates were 87% (11/15) for PGY1, 31% (4/13) for PGY2, and 67% (8/12) for PGY3, and burnout rates were 0% (0/11), 25% (1/4), and 13% (1/8), respectively. PGY1 and PGY3 showed improvement in burnout rates, with a nearly 25% decrease in PGY3 burnout rates after intervention. PGY2 burnout rates worsened after intervention. Almost 25% of residents in each PGY level responded yes to a single question about feeling a great deal of stress on the job, which did not change with intervention. Collection of baseline and postintervention data for the other GME programs at OH is ongoing.

###### Conclusion:

Our project team established, for the first time, a database for burnout rates across all GME programs at our institution. Our results are difficult to interpret without assuming some respondents were not forthcoming with their answers. The postintervention burnout rate of 0 among PGY1 residents and the worsening of burnout among PGY2 respondents contrast with the 25% of residents across all PGY levels/programs responding positively to job-related stress.

**Table:** PROJECT MANAGEMENT PLAN – Wellness at Orlando Health

Vision Statement	Orlando Health as an organization will be a place where all employees, and more so learners, will find balance in personal and professional goals. We aim to create awareness within supportive programs tethered to each branch of our organization and to improve physician burnout rates and develop resilience.
Team Objectives	Our objectives were to decrease physician burnout by 10% within 6 months, and in the process, promote resilience and a joyful working environment.
Success Factors	The most successful parts of our work were establishing a database for burnout rates across all programs in our institution and participating in the development of a website for wellness for the entire organization. We were inspired by GME/DIO support to encourage residents to engage with physician coaches.
Barriers	The largest barrier encountered was skepticism on the part of residents about answering the questionnaire honestly for fear of punitive measures, especially in very small programs. We worked to overcome this challenge by providing reassurance through lectures, champions, small group settings, and a website for GME well-being. Other barriers included challenges in choosing an appropriate tool for assessing burnout stigma of being labeled with a psychiatric diagnosis with some responses deterrent associated with an online survey (fear of lack of anonymity) challenge of identifying the most cost-effective intervention to decrease burnout rates
Lessons Learned	The single most important piece of advice to provide another team embarking on a similar initiative is to educate, engage, and reassure residents/fellows/subjects of the confidentiality of their responses; we learned to not underestimate the degree of skepticism and fear of stigmatization by learners. Lessons learned were utilized to establish baseline burnout data for all programs with ongoing data collection postintervention.

## OSF Healthcare, Peoria, IL

### Mitigating Frustrating Work Factors

#### Rachel Weberman; Crystal Coan; Michelle DeSutter; Anthony Dwyer; Thomas Santoro; Francis McBee-Orzulak

##### 

###### Background:

Burnout among physicians is a widely recognized crisis in healthcare. The following 3 dynamics demonstrate the crisis: (1) 28% of resident physicians experience a major depressive episode during training, (2) 23% of interns experience suicidal ideation, and (3) healthcare executives face an increasingly disillusioned workforce with career burnout rates >50%. To improve physician burnout, 2 approaches are necessary: improving individual physicians' resilience to stress and reducing stressful stimuli that lead to burnout. Most systems are addressing the issue of physician burnout by focusing on resilience training. This approach may be ineffective as we are treating the symptoms of burnout but not the underlying causes. The aim of this project was to develop and implement a methodology to identify and mitigate frustrating work factors that can be scaled across any level of a healthcare system.

###### Methods:

Our plan involved 3 main steps: recruit key stakeholders, survey, and mitigate. For stakeholders, we developed a lean review team and set goals. On the survey, house staff participants were asked “Please describe a frustrating work factor,” and “How frustrating is the work factor you described?” with the following response options: extremely frustrating, very frustrating, moderately frustrating, slightly frustrating, not frustrating. After conducting the survey, we collated and organized identified work factors and prioritized them for mitigation. During the mitigation step, we plan to analyze responses, develop interventions, and administer a follow-up survey.

###### Results:

We received 67 responses from 290 house staff members and identified 130 frustrating work factors grouped into the following 23 categories: parking, equipment, workspace, treatment teams, paging, orders, elevators, culture, visiting residents, consultants, recycling, call, discharge planning, work-life balance, codes, faculty, miscellaneous, cafeteria, EMR, IDRs, emails, workflow, and residency specific. Eighteen specific interventions were identified as areas for action. House staff expressed feelings of being undervalued and reported ineffective communication regarding policies and current efforts.

###### Conclusion:

The initial survey provided various areas to improve house staff well-being and reduce burnout. The primary focus at this point will be to determine an effective mode of communication for house staff and administration to ensure current efforts are understood. The next step of this study will be to follow through on the 18 interventions. Afterwards, a follow-up survey will be sent to house staff to determine the effects of these efforts. We believe this relatively simple process can be easily scaled to any aspect of a healthcare enterprise. Most important, this survey provided insight into the house staff feeling of being undervalued that is likely contributing to burnout and provided a venue to communicate their issues to institutional leaders.

## OSF Healthcare, Peoria, IL

### From Burnout to Resilience: A Feasibility Study to Improve Resident Physician Burnout and Incorporate a Curriculum to Increase Wellness by Fostering Compassion for Oneself and Others

#### Mark Schlotterback, MD; Aviva Whelan, MD; Jean Clore, PhD; Bhavana Kandikattu, MD; Francis McBee-Orzulak, MD; Deborah Disney, MSEd; Crystal Coan, MDA

##### 

###### Background:

Physician burnout is at an all-time high and on the rise. Burnout is defined by emotional exhaustion, depersonalization, and diminished feelings of personal accomplishment. Burnout severity is related to empathy, compassion, prescribing and referral habits, professionalism, and likelihood of making medical errors. One wellness curriculum that can potentially mitigate burnout is cognitively based compassion training (CBCT). CBCT is a secular adaptation of traditional Indo-Tibetan methods for cultivating compassion for the self and others through increased attitudes of impartiality and gratitude. Previous CBCT research has demonstrated improvements in depression, anxiety, biological stress markers, empathic skills, and compassion in various populations, including medical students. Our objective was to assess the feasibility and acceptability of integrating CBCT into a resident wellness curriculum at the University of Illinois College of Medicine at Peoria (UICOMP). Preoutcome and postoutcome measures of stress, depression, anxiety, compassion, well-being, and resilience among resident physicians enrolled in CBCT and those in an attention control group were also collected. Our hypotheses were (1) CBCT could be effectively adapted for and implemented into a variety of physician residency training program curricula and residents would receive it favorably and (2) residents who participated in CBCT would report decreased levels of perceived stress and associated symptoms and improved quality of life after participation.

###### Methods:

CBCT, developed by Dr. Lobsang Tenzin Negi at Emory University in 2004, was designed to cultivate well-being by teaching structured and progressive contemplative exercises. CBCT promotes a sense of closeness and connectedness with others, strengthening compassionate concern while protecting against empathic fatigue and distress. CBCT was advertised via email to all residents, and resident physicians from various UICOMP residency training programs elected to take the training. The training, taught by CBCT-certified teachers/UICOMP faculty, was delivered in 75-minute or 90-minute classes across 8 or 6 weeks, respectively. Times and locations varied to accommodate as many residents as possible. A Qualtrics survey developed by the authors was administered pre-CBCT and post-CBCT to assess feasibility and acceptability. Clinical outcomes were measured using the following self-report measures administered at the same time: Maslach Burnout Inventory (22 items designed to assess occupational burnout), Depression Anxiety Stress Scale-21 (21-item assessment of depression, anxiety, and stress), and Compassion Scale (21-item measure of compassion toward self and others).

###### Results:

The mean age of participants was 28 years, 50% were male and 50% were female, and 67% of participants were either PGY1 or PGY2. Twenty-seven residents enrolled in CBCT. We saw nonsignificant decreases in depression and anxiety as well as increases in compassion post-CBCT, but we saw no changes in levels of burnout. Residents were asked to give feedback on the feasibility/acceptability of the course by rating statements from 1 (strongly disagree) to 5 (strongly agree). Mean scores were 3.9 for “Changed my mind regarding meditation,” 4.1 for “I feel more capable of responding in a healthier and more helpful way to challenges,” 4.3 for “I believe it would be a benefit to me to keep practicing CBCT,” and 4.4 for “CBCT should continue to be offered on a yearly basis to all residents.” When asked what prevented attendance, responses included patient care responsibilities, post-call, rotations, time of day. When asked how useful the guided meditations were, responses included “I LOVE THEM,” and “very helpful, essential.” Additional comments included “I suffer from anxiety & the practices helped ease my mind,” “Self-compassion is the hardest & one I need to cultivate the most,” and “In a short period of time it provided me tools to deal with high stress situations better.”

###### Conclusion:

We found that CBCT was received favorably by residents who elected to take it, but finding an ideal time of day for all residents was difficult. We observed nonsignificant lower levels of compassion, burnout, depression, and anxiety in residents who elected not to take CBCT. We thought this finding could possibly be related to interest in the course. We were limited by a small sample size, attendance, and survey compliance. Because of difficulty with regular consecutive attendance, we intend to offer drop-in meditation sessions covering CBCT topics.

## Our Lady of the Lake Regional Medical Center, Baton Rouge, LA

### Collaborative Task Force to Restore the Joy in Medicine

#### Keith Rhynes, MD; Laurinda Calongne, EdD; Rebecca Horn, PhD; Eva Mathews, MD; Rumneet Kullar, DO; Lauren Mulligan, MD

##### 

###### Background:

Staff physicians and residents at Our Lady of the Lake are diverse in their backgrounds and experiences. The purpose of this project was to address wellness across the system for physicians and residents of all personal and practice backgrounds by gathering and using their input to design wide-sweeping and targeted interventions to achieve our aims: (1) increase wellness knowledge and self-care skills in our physicians and resident physicians, (2) provide wellness resources to our physicians and resident physicians, and (3) create an organizational culture that values and prioritizes physician well-being and spiritual growth.

###### Methods:

We conducted a baseline anonymous online survey, held collaborative sessions with consultants to design a systemwide physician wellness initiative, and began implementation of interventions for the top 3 issues and targeted interventions for specific subgroups of physicians. Survey measures included the abbreviated Maslach Burnout Inventory, depression screener items, open-ended response items, and performance metrics (retention/turnover rate and Press Ganey results). Planned postintervention measures are a follow-up survey and performance metrics.

###### Results:

Fifty-eight percent of physicians and 50% of residents participated in the initiative, and burnout rates of physicians and residents were 46% and 59%, respectively. Input sessions yielded 3 main sources of burnout: (1) organizational factors, involving efficiency of practice and organizational values, (2) work unit factors, involving efficiency of practice, and (3) personal factors, involving work-life balance and resilience. Participants helped design a physician wellness initiative by placing sticky notes on the following categorized poster boards: women in medicine, wellness and work-life balance, practice improvement strategies, resilience, organizational culture and values, technology, and communication. Organizational leaders worked to create a culture of wellness by implementing executive rounding and Schwartz Rounds and worked to improve efficiency of practice by implementing ambulatory Tap 'n Go and communication apps. Task force physicians implemented several interventions during the project. To create a culture of wellness, they implemented professional growth/education sessions, revamped town hall, and standardized huddles. To promote efficiency of practice, they employed medical scribes and installed previsit planning as well as Epic coaching and optimization. Finally, to strengthen personal resilience, task force physicians held a physician wellness fair, physician socialization opportunities, and presentations by national speakers.

###### Conclusion:

We found that no two physicians have exactly the same wellness needs and that the organization must develop flexible initiatives to fully impact all those who need it. To sustain the initiative, task force members and C-suite leadership need to continue taking ownership of components of the wellness initiative. In the future, we intend to grow resources for physicians and residents.

**Table:** PROJECT MANAGEMENT PLAN – Collaborative Task Force to Restore the Joy in Medicine

Vision Statement	In relentless pursuit of better, Our Lady of the Lake will work to enhance the well-being of our physicians and residents by providing them with the tools to better address their personal needs. We will also work to create an organizational culture that values and prioritizes physician well-being and spiritual growth.
Team Objectives	Our objectives were to increase wellness knowledge and self-care skills in our physicians and resident physicians provide wellness resources to our physicians and resident physicians create an organizational culture that values and prioritizes physician well-being and spiritual growth
Success Factors	The most successful part of our work was convincing the C-suite of the importance of physician wellness. We were inspired by the willingness of our physicians to engage with the task force and share their personal wellness concerns.
Barriers	The largest barrier we encountered was unforeseen leadership changes that delayed C-suite decision-making related to the implementation and sustainability of wellness initiatives. Change in culture, particularly at the system level, takes time under any circumstance. We worked to overcome this delay by exercising patience and giving the leaders the time they needed to focus on the change before continuing with our work.
Lessons Learned	The single most important piece of advice to provide another team embarking on a similar initiative is to be as clear as possible on what your measures of success will be before you begin. The project also needs buy-in and commitment from physicians and the C-suite.

## Saint Francis Hospital and Medical Center, Hartford, CT

### Creating a Culture of Wellness

#### Jeri Hepworth, PhD; Rebecca Crowell, PhD; Ashley Negrini, MS; Brian Riley, DO, MPH; Adam Perrin, MD; Kendra Mahoney, MD; Jessica Perez, MD

##### 

###### Background:

Consistent with Saint Francis Hospital and Medical Center's increased focus on wellness and stress management, this project tracked the impact of targeted wellness curriculum education sessions for residents. Our aim was for specific wellness activities and sessions to demonstrate our institution's commitment to resident health and impact resident burnout, resilience, and coping skills.

###### Methods:

A wellness curriculum was collaboratively developed by family medicine and OB/GYN residency leaders, academic affairs leaders, and the chief wellness officer. Joint educational sessions occurred with residents from the two programs bimonthly for 18 months. The curriculum addressed data on physician burnout and specific coping strategies, including mindfulness, resiliency techniques, cognitive flexibility training, self-care activities, and journaling. Curriculum impact was assessed with a brief anonymous questionnaire related to resident wellness, distributed to residents twice yearly, from June 2017 to February 2019. Three dimensions of burnout (accomplishment, depersonalization, and emotional fatigue) were assessed using 3 question subscales from the brief Maslach Burnout Inventory, scored from 0 (never) to 6 (every day). Resilience was measured by the Brief Resilience Scale, scored from 1 (strongly agree) to 6 (strongly disagree), with scores reverse-coded as appropriate. Higher scores indicate more accomplishment, depersonalization, or higher resilience. Overall burnout was assessed by combining depersonalization and emotional fatigue.

###### Results:

Fifty-four OB/GYN and family medicine residents participated between June 2017 and February 2018 (year 1), and 44 between June 2018 and February 2019 (year 2). Of the 98 participants, 76.5% were female, 41% identified as an ethnic minority, and 62.2% were 30 years of age or younger. Maslach emotional fatigue was significantly lower (*P*=0.014) and resilience significantly higher (*P*=0.003) in year 2 compared to year 1. Differences in median Maslach accomplishment and depersonalization subscales were not statistically significant (*P*>0.050). Compared to PGY1 residents, PGY2 residents had significantly higher depersonalization, emotional fatigue, and overall burnout scores (*P*<0.001).

###### Conclusion:

We learned that differently scheduled didactic times impact the feasibility of multidisciplinary training. Further, clinical responsibilities and resident choices about their own wellness impact session attendance. Residents provided feedback that they prefer activities, not just discussion. Examples of activities include providing journals and instruction as well as personal experience of stress-reduction activities. Program directors will continue to collaborate to offer this curriculum in future years. Wellness has become part of our institutional culture, and along with this initiative, other wellness programs and sessions supported our goals. Obtaining resident input during wellness session planning is key, and being flexible with scheduling is critical.

**Table:** PROJECT MANAGEMENT PLAN – Creating a Culture of Wellness

Vision Statement	The AIAMC project will raise awareness and provide specific tools to enhance trainee well-being throughout our institution.
Team Objectives	Our objectives were to assess trainee well-being, develop and implement programs and resources, and advocate for the importance of addressing trainee and physician well-being throughout the institution.
Success Factors	The most successful part of our work was engaging the residents on topics of importance to them and their wellness. It was important for us to get their feedback on what they wanted. We were inspired by the institutional culture change and strong focus on wellness that aligned with our efforts.
Barriers	The largest barriers we encountered were attendance and scheduling sessions that worked well for both the OB/GYN and family medicine residency programs. We worked to overcome these barriers by working closely with program participants, who worked hard to promote and protect time for the sessions.
Lessons Learned	The single most important piece of advice to provide another team embarking on a similar initiative is to include residents in planning sessions to find out what they want.

## Sinai Hospital of Baltimore, Baltimore, MD

### Promoting Resident and Faculty Well-Being – The 3 Ps: Policy, Practices, and Programs

#### Dr. Asha Thomas; Dr. Donald Abrams; Dr. Diane Maloney-Krichmar; Diane Johnson; Tina Gionet; Lucretia Wilson; Dr. Melanie Contois; Dr. En Yaw Hong

##### 

###### Background:

In fall 2016, Sinai Hospital of Baltimore GME established a GME well-being subcommittee to guide the institution's response to the ACGME's revised working and learning environment guidelines and address the growing evidence that medical students, residents, and attending physicians were experiencing burnout at alarming rates. After conducting an inventory of institutional and departmental programs focused on physician and resident well-being, we saw a lack of coordination, focus, and consistency across departments. Our objective was to establish a baseline for burnout among residents and faculty in 6 ACGME residency programs to better understand the work environment and to guide the development of policies, practices, and programs to create an institutional culture of well-being.

###### Methods:

We customized the Areas of Worklife Survey (AWS) + Maslach Burnout Inventory-Human Services Survey (MBI-HSS) to capture program, role, age, gender, and length of service data and invited 149 residents and 72 core faculty to participate. We presented the data for our survey group as a whole, and we will analyze the data by variables and conduct focus group interviews in AY 2020.

###### Results:

The overall participation rate was 56% (n=124); 52% of residents (n=78) and 63% of faculty (n=46) participated in the survey. Compared to 11,000+ people in the human services professions, participant group scores for the 3 MBI-HSS (medical personnel) scales were higher for emotional exhaustion, higher for personal accomplishment, and the same for depersonalization. Participants' ratings of workload, control, reward, community, fairness, and values were similar to the general population ratings. We originally envisioned our project beginning with the administration of the AWS + MBI-HSS, followed by a train-the-trainer program for faculty to provide the tools to integrate resilience training into our residency training programs. We would then administer the AWS + MBI-HSS a second time. Because of several challenges, we were unable to implement the plan. We regrouped and decided to move forward with the AWS + MBI-HSS survey to gain a deeper understanding of the levels of burnout among our residents and faculty.

###### Conclusion:

We learned that bringing about change requires time and effort. Team members often found that their commitments to teaching or administering residency programs took priority over the time needed to implement the project. Stakeholders from other areas often did not have the resources to become fully participating partners. Starting with the AWS + MBI-HSS survey immediately would have been useful. The survey is critical for developing and implementing a plan and assessing the level of resources needed. Dedicated staff and budgeted resources are needed to sustain creating a culture of well-being within the institution.

**Table:** PROJECT MANAGEMENT PLAN – Promoting Resident and Faculty Well-Being – The 3 Ps: Policy, Practices, and Programs

Vision Statement	Sinai Hospital of Baltimore promotes a culture of well-being that supports and strengthens the ability of resident physicians, faculty physicians, and all healthcare team members to grow and thrive in their professional and personal lives.
Team Objectives	Our objective was to develop policies, practices, and programs that equip resident physicians and faculty physicians with resources and practical skills that build self-awareness and flexible thinking, promote regulation of emotions and energy levels, and build connections that promote community at work and improve the clinical and educational environment.
Success Factors	The most successful parts of our work were raising awareness of the complexity of the drivers of burnout and engaging residents, physicians, and the GME community at our hospital in the process of creating working and learning environments to combat burnout and support engagement.
Barriers	Finding a training partner for the faculty train-the-trainer aspect of our project was very difficult. In addition, the time needed to fully implement this project was ultimately beyond the time team members could devote. We worked to overcome this difficulty by scaling back our project to focus on surveying our faculty and residents to determine their levels of burnout and stress. We continued to provide education, training, and support to our residency programs for well-being activities and initiatives.
Lessons Learned	The single most important piece of advice to provide another team embarking on a similar initiative is to either select a small project or obtain a commitment from leadership to assign a staff member full time to the project.

## The Christ Hospital Health Network, Cincinnati, OH

### Creating Camaraderie and Connectedness at Christ Hospital

#### Aurora Rivendale, MD; Gopal Koneru, MD; Alexandra Macpherson, MD; Melissa Mefford, MD; Annette Lewis; Jennifer Reemtsma; John Schroder, MD

##### 

###### Background:

Resident and physician burnout have been highlighted as crises in national news and around the world. Wellness programs and curricula have been implemented to mitigate these effects. Burnout is a product of the demands of the job and the changing nature of medicine in the modern age of the EMR, as well as blurred boundaries between work life and home life. Given this understanding, we chose to focus on ways to build camaraderie, connectedness, and resiliency among peers by providing wellness events and resources. Our objectives were to (1) hold at least 4 wellness events, (2) foster connection and camaraderie between residents and fellows, (3) create a more direct and robust connection between residents and the GME office, (4) create sustainability for future initiatives and have residency committees become subgroups of the GMEC, and (5) provide tools for resilience and resources for wellness.

###### Methods:

All residents and fellows from residency programs at The Christ Hospital were invited to attend the 4 wellness events. Additional interventions included multiple GMEC-sponsored wellness events onsite and off campus, creation of a wellness resource page, and distribution of surveys to assess the needs/wants of residents. We conducted a poll asking about timing, types, and frequency of events, as well as and demographics of class and residency/fellowship at the first event and sent out a midyear survey. We also asked for in-person feedback at meetings with residents and at the events. The response to the initial poll was good; the response to the survey was limited. We chose not to do a formal research study.

###### Results:

We hosted 4 events, 3 of which were on campus and had specific focuses. A winter gala held off campus in January was a success. Thirty individuals RSVPed, and at least 47 residents and spouses came. Twenty-eight participants responded to the poll and requested the following activities: sand volleyball at Fifty West Brewing (27), group laser tag (24), yoga classes at Christ on the rooftop (16), group classes such as cooking, dancing, and exercise (16), and other (16). Eighteen participants responded to the survey. The survey asked, “Did you attend an event?” “Did it benefit your wellness/camaraderie?” and “What would you like in the future?” Thirteen participants said they attended an event, and 5 said they did not. Respondents did not report any negative comments, and 76% found that the event increased wellness. One participant commented, “I can honestly say that over my three years as a resident here, the interaction with and camaraderie with residents from the other programs here at Christ has improved [thanks] to all the recent wellness events and I am very grateful for the institutional support. In summary, thank you for supporting our residency wellness.” Another participant commented, “I feel like the events we have now are very well put together and help the morale out a lot.”

###### Conclusion:

Qualitatively, residents and staff both reported an increased sense of camaraderie and connectedness. We received several responses that the events were well received and well appreciated. Wellness committees are now integrated into GMEC. When planning the next event, we will incorporate requests from residents and fellows and look for outside staffing for yoga/dance classes. We have requested onsite gym facilities and concierge services. In the future, we will ideally have a position within the GME office that focuses on wellness to help maintain continuity for years to come. We will continue to discuss wellness initiatives during yearly retreats and request feedback from residents.

**Table:** PROJECT MANAGEMENT PLAN – Creating Camaraderie and Connectedness at Christ Hospital

Vision Statement	We recognize the individual nature of each person's whole health. As physicians, we hope to be models for our patients, and we hope to care for ourselves so we can best serve others. We as a group will strive to foster a community and culture that are diverse, supportive in times of adversity, connected, and reaffirming of each person's goals and dreams. Wellness is multifaceted, as is burnout. We envision providing both the intangible and tangible resources needed to maintain wellness and resilience during the challenging period of residency and beyond. Many things are beyond one's control in residency, but where possible we will foster peer connection, a sense of meaning in one's work, recognition of our success, and resilience in the day-to-day struggles. As a group, we intend to create events that span the different residency programs. These events will serve many functions and be composed of stress-reduction activities such as yoga and mindfulness, as well as social events and community service. The aim is to bring us together as a larger community. These events will be periodic and scheduled so as many people as possible can attend over time. Our hope is that by connecting the different programs we will have a better sense of collegiality and camaraderie at work which will aid in clear communication, positive morale, and excellent patient care. We also envision creating a site that will provide resources for wellness in the community including places to eat, religious communities to attend, athletic pursuits, and family activities, as well as mental and physical health recommendations.
Team Objectives	Our objectives were to create onsite events that bring different residencies and fellowships together and provide resources about health and mental counseling. These objectives require funding and some organization from both the GME office and residents in each class.
Success Factors	The most successful part of our work was seeing more residents engaging with one another outside of these events in the cafeteria and on the floors. We received positive feedback and requests for new and different events. We were inspired by other residencies in the system and what they have already started doing to inspire and foster wellness in their residents.
Barriers	The largest barriers we encountered were lack of time and energy organizing events, getting event organizers together, and getting the residents to attend events. We worked to improve attendance by having multiple reminders at meetings by residents who were involved. Word of mouth seemed the best way to motivate people to come to events. We also sent official invites for the event that was off campus to which people RSVPed appropriately. Another barrier we faced was difficulty obtaining clear feedback with low yield on surveys, likely because of email/survey fatigue from residents.
Lessons Learned	The single most important piece of advice to provide another team embarking on a similar initiative is to have a team leader, delegate roles early on, and have people stick to their roles. Recognize that residents' schedules are chaotic, and having a point person in the GME office to focus on planning and organizing events is very useful. We were lucky to have someone willing to do that.

## TriHealth, Cincinnati, OH

### Physician Collegiality and Wellness

#### David Dhanraj, MD, MBA; Elizabeth Beiter, MD; Kimberly Bethea, MD; Rachel Bramblet, DO; Becky Fleig; Neha Gandhi, MD; Kevin Grannan, MD; Laura Hampel, MD; Michael Holbert, MD; Helen Koselka, MD; Michelle Lopez, MD; John Mitko, MD; Chadd Todd, MD; Peter Tran, MD; Barbara Wexelman, MD; Erin Worden, MD; Muhammad Zubair, MD; Steven Johnson, MD; Erin Maetzold, MD

##### 

###### Background:

TriHealth is in the midst of a transformation in culture including the way physicians interact with and lead our healthcare teams. To support this transformation, we aim to focus on improving physician wellness, collegiality, and engagement. The TriHealth residents identified that lack of communication and collegiality during the consultation process was contributing to burnout and that improving this process could contribute to wellness. Our objective was to implement a process in GME that would measurably improve collegiality and satisfaction in the consultation process. We believed this initiative would improve patient care and model behavior for our entire medical staff. The project would include improvements to our EMR and information systems to facilitate physicians finding one another and engaging in a collegial interaction.

###### Methods:

We developed a consultation compact and a consult checklist that included the following steps: (1) review the on-call list for the specialty, (2) contact the consultant directly, (3) include the name and number of the referring physician on the consult or the number of the person on call if the referring physician is signing out shortly, (4) state clearly the reason for the consult, (5) note the urgency of the consult, (6) include a direct contact number or pager of the consultant at the bottom of the note (ideally not an office number). A consultation survey, which we intend to repeat every quarter, was distributed to residents and faculty attendings from family medicine, internal medicine, OB/GYN, and surgery. The consultation survey uses a 5-point Likert scale and includes the following items: (1) How satisfied are you, overall, with the consultation process? (2) Direct provider-to-provider communication occurs during consultation process; (3) The interaction between providers during the consultation process is collegial; (4) Consultants clearly addressed the clinical question for which they were consulted; (5) Consultants provided appropriate care for my patient; (6) Consultants provided care within an appropriate time frame. In addition, an inventory of program-specific wellness activities was shared, and a yearly physician wellness fair, featuring system resources for physician wellness, was implemented. Finally, we represented TriHealth in our citywide coalition for physician wellness.

###### Results:

While the overall satisfaction level with the consultation process appears to be good, a large portion of both consultants and consulting physicians reported low levels of direct interaction. Based on initial screening, consulting physicians appear to have a generally higher level of satisfaction than consultant physicians regarding the overall consultation process. We have started a collaboration with the ICU to deploy our consultation compact outside of GME, and we are planning to make the resident wellness fair an annual event. Program inventories of wellness activities were completed and will be annually updated, and a GME wellness subcommittee was formed to develop common program initiatives around wellness.

###### Conclusion:

Collegiality and effective communication are key drivers for wellness/burnout for resident physicians at our institution. Improving the overall consultation experience without the adoption of the process outside of GME was difficult, and we found significant variation among programs in how they address wellness. Moving forward, we will continue to measure the consultation process quarterly. We will work toward partnerships with physicians and departments outside of GME, present our results to the TriHealth Patient Care Committee for hospitalwide implementation, and continue the yearly wellness fair. We plan to integrate the EAP/behavioral health into the curriculum to normalize use of these services. Our CEO and CMO have instituted an effort toward reducing error, improving quality, and having all physicians function as members of one system. Improving communication among physicians is now a system goal. A new communication platform was launched, but it does not address the need for accurate information about available/on-call colleagues for clinical collaboration. Our GME programs are willing partners for developing effective use of technology and determining how to address the needs of our system.

**Table:** PROJECT MANAGEMENT PLAN – Physician Collegiality and Wellness

Vision Statement	We envision TriHealth being a place where our physicians (residents, faculty, attendings) come to work feeling energized, refreshed, and engaged in our overall mission of patient care. We want to create a healthcare environment that can identify factors associated with physician frustration and burnout and that works systematically to reduce or eliminate these factors, knowing such efforts have been shown to improve patient outcomes.
Team Objectives	The project required development of a consultation compact with expected behaviors for all of the physicians in GME as well as inventories of current wellness initiatives from each program. Stakeholders include representatives from all residency programs, GME and CMO leadership, and IT. Our participation in this initiative will help us move toward a culture that promotes physician well-being, a supportive work environment, decreased physician turnover, and improved patient outcomes.
Success Factors	The most successful part of our work was finding consensus among the resident representatives and faculty around the need to improve the consultation process to reduce burnout and improve satisfaction in the work of patient care. We were inspired by the stories, both good and bad, about how collegiality (or lack thereof) affected patient care.
Barriers	The largest barrier we encountered was that much of the frustration and difficulty in the consultation process originated from physician staff outside of GME. To overcome this obstacle, we found a physician leader late in the project who was independently working on a similar project in his department. We agreed to work together to establish our compact in the ICU. Other limitations included the lack of ability to give feedback during positive and negative interactions and the systemwide tool for evaluating wellness being transitioned by our organization midproject.
Lessons Learned	The single most important piece of advice to provide another team embarking on a similar initiative is that when your project coincides with a systemwide change you intend to be part of your work, make certain that the timelines being promised by your organization are realistic and fit the timeline of your project. A large portion of our project was dependent on platforms that were delayed in their deployment by our organization. If you are considering a project that involves making a significant change (especially behavioral) in your organization, enlisting key stakeholders from outside GME to actively participate in your project is critical.

## UnityPoint Health – Des Moines, Des Moines, IA

### Developing and Improving an Institutional Approach to Resident Well-Being

#### Catherine H Renner; Elizabeth A Bolten; Chanteau Ayers; Hayden L Smith; Maheen Shakoor; Mohamed Elfeki; Hope Villiard; William J Yost

##### 

###### Background:

Six community hospital-based residency programs with varying numbers of residents—family medicine, internal medicine, general surgery, pediatrics, podiatry, and transitional year—have no institutionally regulated policies on monitoring and guiding resident wellness. Programmatic activities to address resident wellness exist with no common institutional approach, and we noticed a lack of clear-cut institutional support for and commitment to the well-being of residents. Our objectives were to (1) educate residents on self-assessment tools to use during residency and independent practice, (2) survey residents to measure wellness at regular intervals, (3) develop a GMEC subcommittee to lead the development of institutional policies that provide a systematic approach for measurement of wellness and interventions to assess and address resident wellness, and (4) have the GMEC subcommittee inform institutional strategy on resident well-being activities and develop a core curriculum.

###### Methods:

We recruited residents from the six residency programs, distributed four surveys, and studied one focus group for more than 2 academic years. Survey completion was optional, and responses were reported by program year. During the second year of our project, we implemented a core conference curriculum that allows the institution to provide well-being tools and resources to residents throughout the academic year with the aim of addressing/minimizing well-being issues. We also created a GMEC wellness subcommittee comprised of institutional leaders, faculty, residents, and the employee assistance director. We initially measured depression, dependence, exhaustion, and burnout using the Maslach Burnout Inventory (MBI), CAGE (cut-annoyed-guilty-eye) questionnaire, Epworth Sleepiness Scale (ESS), and Zung Self-Rating Depression Scale. Subsequent surveys were shortened, eliminating the ESS and CAGE questionnaire. We attempted to facilitate 6 focus groups of 6 to 10 residents each between the second and third surveys. Only one focus group was successful, and the results mirrored the survey. Therefore, we decided not to repeat the focus groups as a measurement method. We issued the surveys in June 2017, December 2017, May 2018, and December 2018.

###### Results:

We generally had high response rates for all surveys, although rates began to decline toward the end of the project. Although our objective was to provide residents tools to measure burnout, stress, depression, and exhaustion, resident awareness of this objective declined with each survey iteration. Resident perceptions of program support had higher response rates for “strong” and “very supportive” compared to “nonexistent,” “mediocre,” and not reported/blank. Results for depersonalization and emotional exhaustion were corroborative.

###### Conclusion:

Addressing resident well-being is a complex and multifactorial task that may benefit from small incremental changes. Multiple efforts have been made to embed well-being actions into medical education, expanding to include faculty, staff, and medical students. Mental wellness as a concern is not restricted to GME and should be an organizationwide priority.

**Table:** PROJECT MANAGEMENT PLAN – Developing and Improving an Institutional Approach to Resident Well-Being

Vision Statement	By obtaining and analyzing mental wellness of residents in each program, we hope to establish interventions that aid in the reduction of depression, sleep deprivation, and other stressors. We also intend to educate residents on the tools available for self-assessment to recognize depression, dependence, and sleep deprivation. With this project, we attempted to provide mechanisms for residents to handle these issues.
Team Objectives	To address mental health and wellness in residents, we aimed to develop institutional interventions that could be planned as regular activities and used by programs in which residents are known to have these issues. We planned to educate residents on the tools available for self-assessment to prepare them for when they are practicing independently.
Success Factors	Two successes resulted from this project: the creation of the GMEC wellness subcommittee and the development of a wellness core conference curriculum. The committee is comprised of residency faculty, residents, GME leadership, medical school leadership, and an employee assistance representative. The wellness core conference curriculum is a series of lectures created to address common issues/stressors identified through surveys. These lectures are open to all residency programs and recorded for those who are not able to attend. We were inspired by the comments provided by responders, giving some insight into the challenges residents face. We were also inspired by the engagement of faculty in addressing these issues.
Barriers	Project evaluation and lack of time were the largest barriers. Finding the time needed between collecting and analyzing data from surveys and implementing interventions was a challenge, and determining if an intervention directly affected the data from the follow-up survey was difficult. We are strategizing on how to assess interventions in proximity to the implementation, which would allow us to better assess correlation. Another limitation was developing institutional surveys that were voluntary, self-reported, and analyzed to create one-size-fits-all interventions distributed across programs.
Lessons Learned	Communicating results to residents is essential to the success of survey longevity. We saw from repeated surveys that some residents are not aware of interventions implemented as a result of the initiative. We think continuing this dialog and ensuring that we are connecting the dots of the survey/intervention relationship are important.

## Virginia Mason Medical Center, Seattle, WA

### Creating a Culture of Resident Well-Being: Access, Support, and Connection

#### Jeffrey Rouse, MD; Meriah Moore, MD; Sarah Nobles, MD; Alex Ajeto, MD; Kelly Hendershot, MD; Nicketti Handy, MD; Alvin Calderon, MD; Ryan Pong, MD; Jennifer Richards; Joyce Lammert, MD; Gillian Abshire, RN & TL

##### 

###### Background:

Virginia Mason Medical Center's 2017 CLER review showed that 61% of residents would power through the end of their shift, and only 28% of residents would notify their supervisor of fatigue. Compelled by these findings, a team comprised of residents, faculty, and GME leaders identified 3 domains for improvement: (1) Make it easier for residents to ask for help and provide support when they do; (2) Improve access to resources; and (3) increase connectedness. Improvement work in these areas supports one of Virginia Mason's current organizational goals, “Respect for People,” and the GME vision to shape the future of healthcare by transforming medical education. We categorized the project's 8 initiatives into 3 domains: Access to Resources (access and awareness of benefits, streamlining EAP access, and time off); Asking for Help (1-to-1 meetings, ICU death rounds, and wards tournament); and Creating Connections (multidisciplinary rounds and social events).

###### Methods:

To improve access to resources, we administered a resident survey, improved website tools and awareness of EAP services, and defined sick/leave/vacation time-off policies by program. To make it easier to ask for help, we implemented biweekly intern-to-intern groups, monthly death rounds in the ICU, and wards tournaments. To increase connectedness, we instituted monthly grief pauses in the critical care unit and added multidisciplinary resident-led rounds. Examples of metrics we planned to monitor were the following: improved sense of teamwork (survey, target: 50% positive responses postintervention), death rounds (residents report personal benefit, target: 50% improvement postintervention), awareness of EAP benefits (process metric, target: EAP flyer created and distributed), and availability of sick/leave/vacation policies (target: 100% of programs have policies written and available online).

###### Results:

Preliminary data obtained from pulse surveys were as follows: 87% of respondents reported feeling supported by other residents (up from 84%); 66% reported pride in their work (down from 80%); 58% reported vitality to do work (down from 74%); 38% were eager to return to work each day (down from 66%); and 32% felt they make the world better (down from 65%). Intern-to-intern groups are facilitated biweekly. After participating, interns reported a 32% increase in resiliency and a 42% increase in morning energy levels. Death rounds are facilitated monthly. Sixty percent of senior residents have participated and completed experience-based design surveys, and 85% of responses reflected positive personal benefit from the intervention. Two multidisciplinary rounds were held, and improvements and adjustments were made from first to second PDSA. We are planning to retest with smaller groups focusing on two programs each session. Organizationwide change is underway as a result of the 8 initiatives. Examples of change include increased awareness of the organization's EAP physician/resident-tailored services and implementation of Auto-Pause (triage, replacement from duties, recover) for when emotionally taxing care events occur. Our big dot metric, the ACGME survey, was expected in May 2019. Once received, the final work is to analyze results, apply learnings to each of the initiatives, and make adjustments to the processes in place to improve outcomes.

###### Conclusion:

Survey results may differ based on venue vs seasonal variations (ACGME survey in the summer vs pulse surveys in the fall/winter). Moving forward, we will maintain the GME website, increase awareness of available benefits, and increase awareness of EAP benefits for residents. Our third PDSA in fall 2019 will be a multidisciplinary learning event involving two residency programs at a time rather than every program at once. The PDSA is to build sustainable, resident-centric opportunities for fun. Resident wellness and resilience improvements at Virginia Mason have made significant initial strides and will always be part of the conversation when considering the health and well-being of our GME programs as we move into the future. Improving the culture of well-being and building lifelong resilience among residents and faculty will require a multimodal approach that will need sustained work in the coming years.

**Table:** PROJECT MANAGEMENT PLAN – Creating a Culture of Resident Well-Being: Access, Support, and Connection

Vision Statement	Our vision is to inspire others to work toward respect, support, and wellness for all people.
Team Objectives	The following hallmarks guided this project's aims: Trust and support define the relationship among residents and faculty. There is no hesitation to ask for help in the GME program. When help is needed, it is available. The processes for how to support residents are transparent, fair, and well understood by residents and faculty. Residents feel connected to their purpose in medicine.
Success Factors	The most successful part of our project was realizing that the work of the residents was impacting other target populations within the organization (providers and multidisciplinary teams). For example, the deep dive into understanding a process which had been created but had fallen into latency resulted in the reignition of that process and its spread to also apply to providers (S.O.S. became the topic of a rapid process improvement workshop that resulted in Auto-Pause). We were inspired by the receptivity of our multidisciplinary team members to partner with us to tackle the issues, seek solutions, and try a few things (eg, intern-to-intern social workers, wards tournament with hospitalists and medical students, and death rounds with the palliative care attending).
Barriers	The largest barrier we encountered was lack of designated time for residents to work together in teams. Offsite rotations take valuable team members offline for large chunks of time. We worked to overcome this barrier by having multiple team members share the tasks through handoffs and selecting team members who had at least 2 to 3 years left in their tenure at the organization. Other limitations included competing priorities, as well as difficulty operationalizing resources, freeing the funds, and providing leadership for management (eg, a social worker for intern-to-intern groups).
Lessons Learned	The single most important piece of advice to provide another team embarking on a similar initiative is to trust the process and make small incremental improvements, steadfastly with joy and patience. Also, use your existing internal tools.

